# π-Extended benzo[1,2:4,5]di[7]annulene bis(dicarboximide)s – a new class of non-alternant polycyclic aromatic dicarboximides†

**DOI:** 10.1039/d3sc04015a

**Published:** 2023-09-20

**Authors:** Jonas Spengler, Chongwei Zhu, Kazutaka Shoyama, Frank Würthner

**Affiliations:** a Universität Würzburg, Institut für Organische Chemie and Center for Nanosystems Chemistry Am Hubland 97074 Würzburg Germany wuerthner@uni-wuerzburg.de

## Abstract

Aromatic dicarboximides are a class of molecules represented by the well-known rylene bis(dicarboximide)s, in particular perylene or naphthalene bis(dicarboximide)s, which show pronounced optoelectronic properties and are applied as color pigments, fluorescent dyes and organic semiconductors. Herein we extend the family of aromatic bis(dicarboximide)s and report the synthesis of the first series of non-alternant aromatic dicarboximides by twofold Pd-catalyzed [5 + 2] annulation. Characterization by UV/vis spectroscopy and cyclic voltammetry (CV) measurements give insight into the optoelectronic characteristics of the hitherto unexplored substance class of heptagon-containing imides. Theoretical studies by nucleus independent chemical shift (NICS) XY-scans and anisotropy of the induced current density (ACID) plots demonstrate the influence of both the non-alternant carbon framework and the imide moieties on aromaticity of the synthesized bisimides.

## Abstract 2

A series of non-alternant aromatic dicarboximides was synthesized by manipulating the perylene bisimide framework through the introduction of heptagonal rings, which strongly alter their optoelectronic properties.

## Graphical abstract

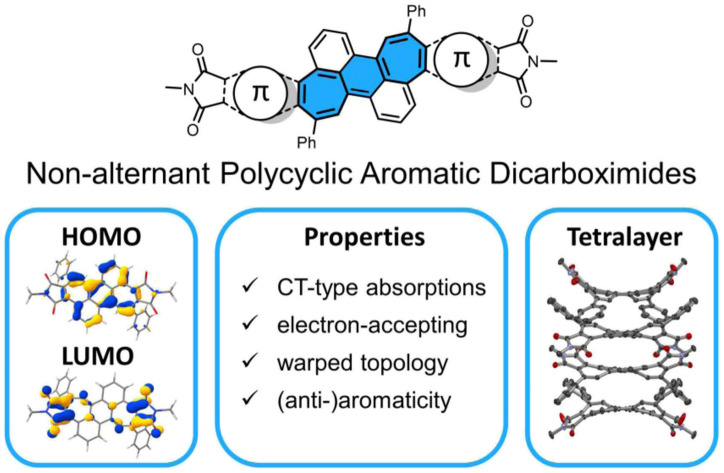

## Introduction

**Fig. 1:**
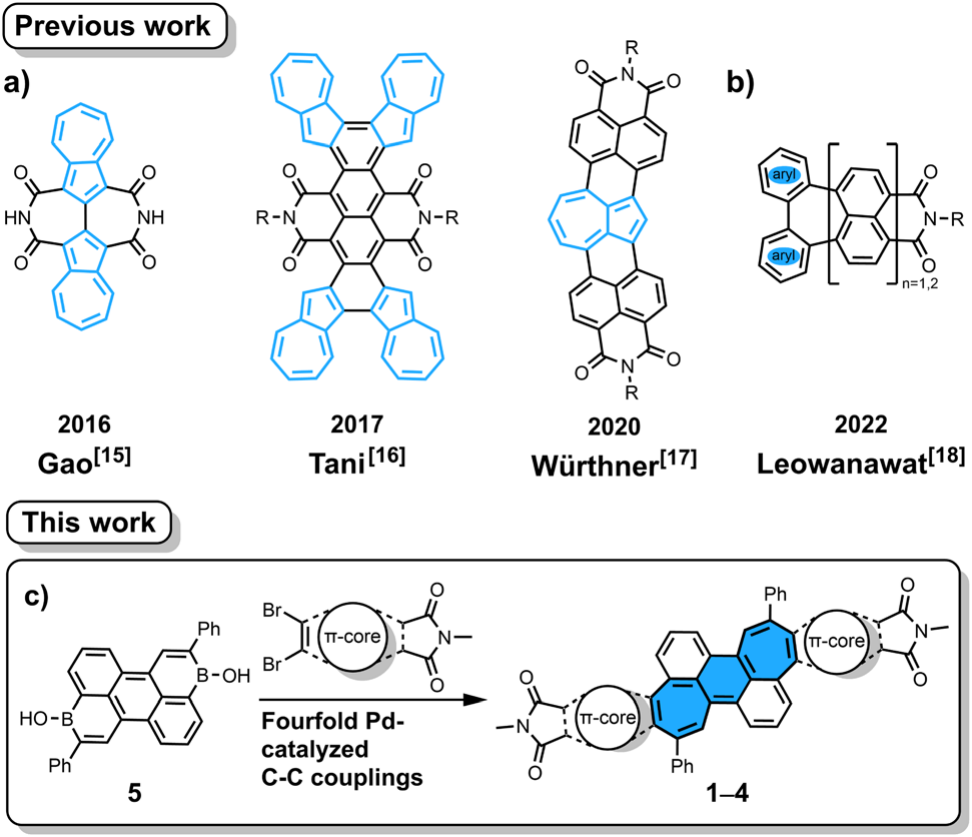
Reported non-alternant imides synthesized by azulene annulation (a) and by a decarboxylative [3 + 4] annulation protocol (b). Pd-catalyzed [5 + 2] annulation (c) to give non-alternant bisimides 1–4. The structure of benzo[1,2:4,5]di[7]annulene is highlighted with blue filling in (c). R = *n*-Bu/*n*-Oct.

Imide-functionalized polycyclic aromatic hydrocarbons (PAHs) have attracted tremendous attention in various fields including supramolecular chemistry and materials chemistry due to their intriguing optoelectronic properties.^1–9^ Specifically, attachment of imide groups at PAHs affords improved coloristic properties (absorption at longer wavelength with higher molar absorption coefficient) as well as lower-lying frontier orbitals. The latter effects are important to change semiconducting properties from p- to n-type and to increase the photostability of colorants. In contrast to the widely studied rylene-based mono- or bis(dicarboximide)s, their non-alternant counterparts,^10^*i.e.* those containing odd-membered rings, are underdeveloped.^3–7^ Introducing odd-membered rings into the hexagonal carbon skeleton of PAHs induces unique properties such as large dipole moments, increased fluorescence lifetimes, biradical character and high electron affinities.^11–14^ The difficulties in preparing such non-alternant aromatic imides stem from the lack of synthetic methods to connect non-alternant aromatics and imide moieties. There are mainly two approaches known in the literature for such PAHs. The first is annulation of azulene, an archetypical non-alternant building block, onto imide-containing arenes (Fig. 1a).^15–17^ The second is recently developed decarboxylative [3 + 4] annulation of rylene bis(dicarboxylic anhydride) with diphenyliodonium salt, in which a seven-membered ring is formed (Fig. 1b).^18^ The former is applicable only for azulene-containing moieties and the latter requires corresponding rylene bis(dicarboxylic anhydride) as the starting material. Therefore, we contemplated a new more general method to annulate an aromatic imide and another aromatic building block. We conjectured that our recently developed [5 + 2] annulation protocol using aromatic borinic acids and aromatic dibromides^19–21^ would enable direct attachment of dicarboximide onto a seven-membered ring, which then would lead to molecules with remarkable optoelectronic properties.

Herein, we report the synthesis of non-alternant dicarboximides by [5 + 2] annulation^20^ of arene borinic acid 5 and a variety of dibromo-dicarboximides (Fig. 1c). We achieved successful annulation with maleimide, phthalimide, 2,3-naphthalimide, and acenaphthalimide. With maleimide as the imide coupling partner, we synthesized for the first time an aromatic imide molecule with a seven-membered ring directly attached to the imide five-membered ring. This cycloheptaimide derivative showed a charge-transfer type S_0_ → S_1_ transition, a rare property for aromatic imides, which is in stark contrast to the bisimides synthesized with phthalimide or naphthalimide. The aromaticity of these non-alternant bisimides was systematically investigated by NICS and ACID calculations. The insight obtained by our study contributes to designing aromatic imide molecules with tailor-made optoelectronic properties.

## Results and discussion

### Synthesis

**Scheme 1:**
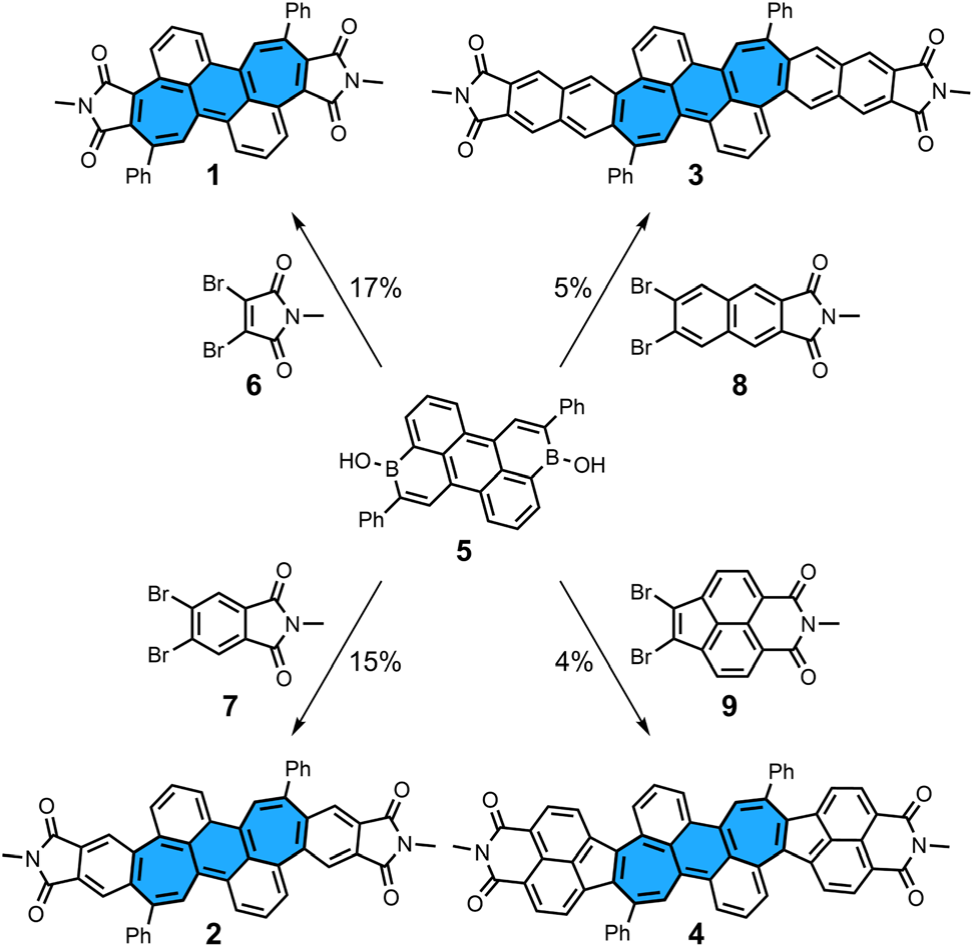
Synthesis of non-alternant bisimides 1–4 and their respective dibromo precursors 6–9. Conditions: dibromo imide (3.0 equiv.), [Pd_2_(dba)_3_]·CHCl_3_ (10 mol%), (^*t*^Bu)_3_P·HBF_4_ (24 mol%), Cs_2_CO_3_ (6.6 equiv.), H_2_O (40 equiv.), ^*t*^AmOH, 100 °C, 65 h. Bu: butyl; dba: dibenzylideneacetone.

We first describe the synthesis of dicyclohepta[*de*,*kl*]anthracene-4,5:11,12-bis(dicarboximide) 1, the smallest molecule in the series presented here (Scheme 1). The starting material, diborinic acid 5, was prepared by the one-pot hydroboration/C–H borylation/dehydrogenation reaction sequence previously reported by our group.^22^ Reacting three equivalents of dibromo-*N*-methylmaleimide 6, one equivalent of diborinic acid 5, [Pd_2_(dba)_3_]·CHCl_3_ as a palladium source, (^*t*^Bu)_3_P·HBF_4_ as a ligand, Cs_2_CO_3_ as a base, ^*t*^AmOH as a solvent, and H_2_O as an additive for 65 h gave the desired bisimide 1 in 17% yield. In contrast to application of the annulation protocol in our previous work,^19–21^ slightly harsher reaction conditions were necessary to obtain a full conversion of starting material. π-Extended derivatives 2–4 could also be prepared by using dibromo precursors 7–9 and similar reaction conditions as for 1 in yields of 4–15% (see ESI† for details and synthesis of precursors 7–9). Attempts to use non-alkylated imides such as dibromomaleimide and dibromophthalimide failed, indicating that *N*-protection is crucial for a successful synthesis. All bisimides were stable under ambient conditions in the solid state and in aerobic solution in the dark, showing no significant decomposition by ^1^H NMR after storage for weeks. However, signs of decompositions were observed within days when azulene-containing bisimide 4 was dissolved in aerobic solution under ambient light. Despite the large π-surfaces all bisimides were highly soluble in halogenated organic solvents as well as non-halogenated organic solvents such as acetone, tetrahydrofuran and ethyl acetate.

### UV/vis spectroscopy

**Fig. 2:**
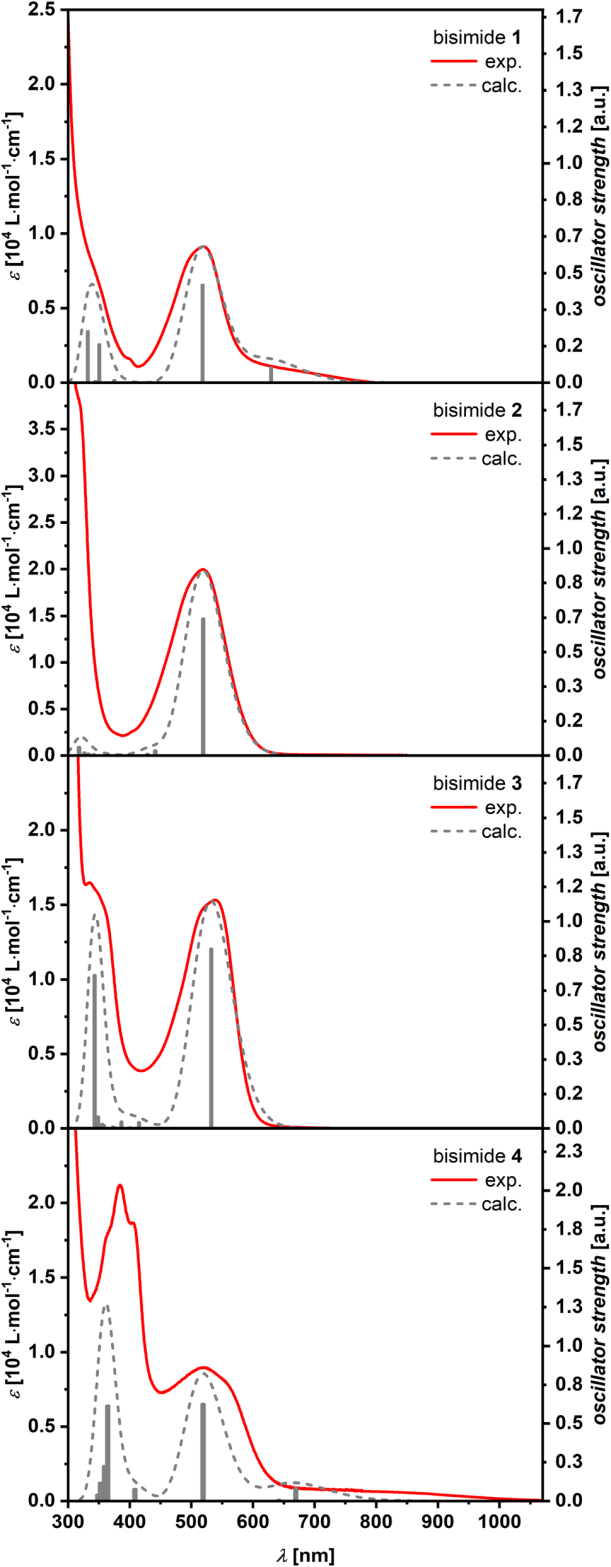
Comparison of the experimental (red solid) and TD-DFT calculated (grey dashed, CAM-B3LYP/6-31+G(d)) UV/vis spectra of bisimides 1–4. The calculated spectra were shifted towards higher energies (1: 820 cm^−1^; 2: 540 cm^−1^; 3: 750 cm^−1^; 4: 820 cm^−1^), scaled (1: 0.27; 2: 0.40; 3: 0.24; 4: 0.24) and convoluted with a phenomenological Gaussian function with a FWHM of 290 cm^−1^.

The optical properties of the new bisimides 1–4 were studied by using their methylene chloride solutions (Fig. 2). Their longest-wavelength maxima were in the visible region at 518–542 nm, which account for the dark red to violet color of all compounds. The absorption spectra of bisimides 1 and 4 showed a shoulder extending to 800 nm for 1 and 1000 nm for 4. These weak absorption bands could be attributed to a charge transfer (CT)-type transition according to DFT calculations (*vide infra*). On the other hand, spectra of 2 and 3 showed more intense peaks at 519 nm (20000 M^−1^ cm^−1^) for 2 and 540 nm (15300 M^−1^ cm^−1^) for 3, respectively, indicating allowed S_0_ → S_1_ transitions. No fluorescence could be observed for 1–4. In the absence of time-resolved spectroscopy we can only speculate that either fast non-radiative relaxation processes *via* conical intersections or efficient intersystem crossing into the triplet manifold lead to a fast decay of the excited state. The former process is often related to structural changes in the excited state^23^ whilst the later process is favored by contortion as exemplified by the fullerenes.^24^

### TD-DFT calculations

**Fig. 3:**
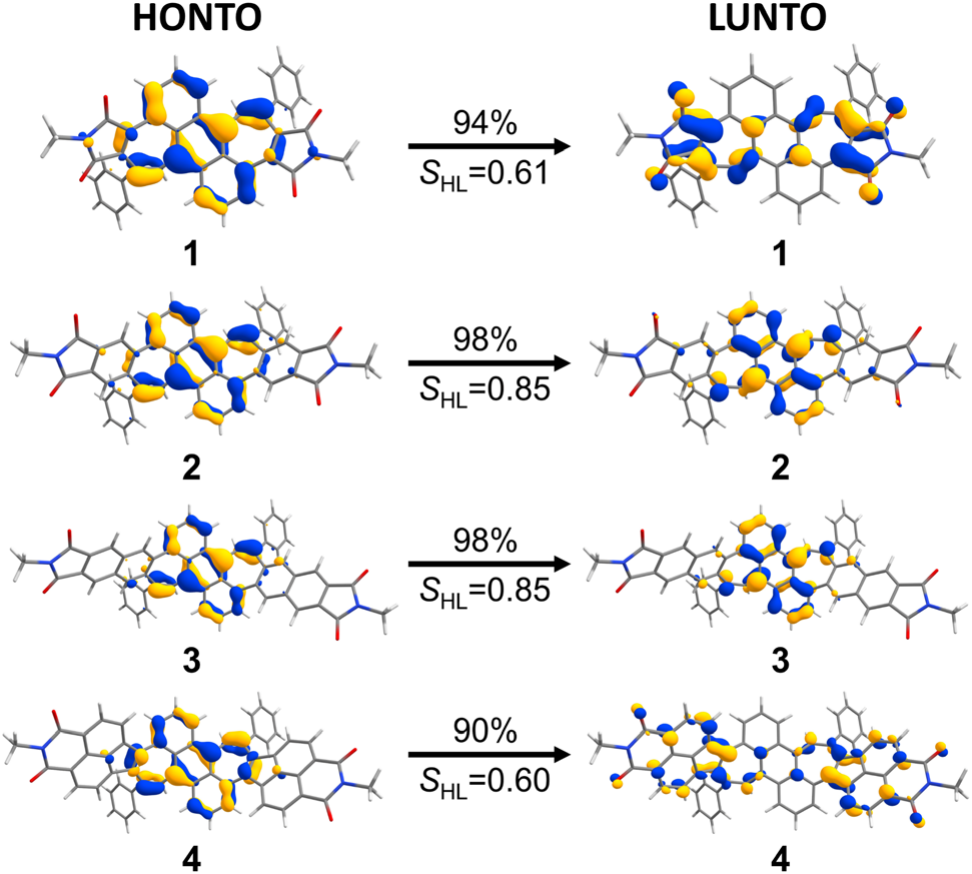
NTO-representations^26^ (isovalue 0.04) of the S_0_ → S_1_ transition as HONTOs (highest occupied natural transition orbitals) and LUNTOs (lowest unoccupied natural transition orbitals) of bisimide 1–4. Corresponding *S*_HL_ values are shown below the arrows. Calculated at the CAM-B3LYP/6-31+G(d) level of theory.

In order to obtain deeper insight into the UV/vis absorption spectra, (TD-)DFT calculations were conducted at the CAM-B3LYP/6-31+G(d) level of theory.^25^ The calculated energies and oscillator strengths for the S_0_ → S_1_ transition of bisimides 1–4 matched well to the observed spectra of them (plotted visually in Fig. 2, see ESI Table S3† for details), demonstrating that the chosen functional and basis set are appropriate for the systems investigated here. The natural transition orbitals (NTOs) provide an intuitive orbital representation of a given electronic transition.^26,27^ In the case of bisimide 1, the highest occupied natural transition orbital (HONTO) is evenly distributed over the π-system, while its lowest unoccupied natural transition orbital (LUNTO) is mainly located at the imide moieties as well as the heptagonal rings (Fig. 3). Such a separation of HONTO and LUNTO indicates CT character of the given transition. The degree of CT character can be gauged by the *S*_HL_ value, which describes the overlap integral of the norm of the HONTO and LUNTO.^28^ The value of *S*_HL_ varies from 0 to 1 where higher values indicate lower CT character and lower values higher CT character. For bisimide 1 a low *S*_HL_ value of 0.61 was calculated (Fig. 3). On the contrary, *S*_HL_ calculated for the non-imidized parent molecule (1a, see Fig. S18† for its structure) was higher than that obtained for bisimide 1 with 0.74 (ESI, Table S2†), verifying that the CT-type transition of 1 arises from the spatial separation of HONTO and LUNTO caused by imide moieties adjacent to heptagonal rings. Further π-extension of 1 by benzene (for 2) or naphthalene (for 3) linker leads to the disappearance of this effect, since both HONTO and LUNTO are now located at the central carbon skeleton and can clearly be assigned to a locally excited (LE) state with a high *S*_HL_ value of 0.85. Introduction of a non-alternant linker (acenaphthene) again increases the CT-character for the longest-wavelength absorption band of bisimide 4 with an *S*_HL_ value of 0.60. While its HONTO, like for the other bisimides 1–3, is located at the central anthracene and heptagonal rings, the LUNTO is mainly located at the acenaphthylene moiety and only to a minor extent at the imide moieties. Therefore, the CT character arises between the central anthracene moiety as the donor and the peripheral azulenonaphthalene moiety as the acceptor.^15–17,29^ These observations show differences in the impact of imide groups to the CT character of 1 and 4. The imide groups of 1 are the sole constituent of the acceptor moieties as indicated by the high orbital coefficients in the LUNTO representation. On the other hand, those of 4 build an acceptor moiety together with the neighboring acenaphthalene units, as seen in the delocalized LUNTO around both the imide and acenaphthalene moieties.

### Aromaticity

Aromaticity is a key property for non-alternant PAHs.^11,14,30,31^ Therefore we investigated the impact of imide groups on the aromaticity of parent PAHs using NICS calculations.^32^ Because there have been multiple cases where the initially proposed method for NICS values lead to misleading or wrong results,^33–35^ we corrected the NICS calculations with the σ-only model for a proper classification of induced ring currents.^36^ Its idea is to account for all the σ-contaminations by calculating the NICS values of the analogous molecule in which the π-system is “switched off” by replacing all the C–C π-bonds by two C–H bonds perpendicular to the π-system (ESI, Fig. S19,† right). Subtracting these contributions of the σ-skeleton to the chemical shielding from the NICS_*zz*_ values of the unchanged molecule (ESI, Fig. S19,† left) results in NICS_π,*zz*_ values entirely caused by π-electrons and free of σ-contaminations. Since the tropicity of multi-ring systems cannot be reliably assessed by only one NICS probe per ring, a NICS-XY-scan was performed by using multiple probes placed 1.7 Å over the π-system connecting linearly the centers of the rings of interest (ESI, Fig. S19†).^36–39^

**Fig. 4:**
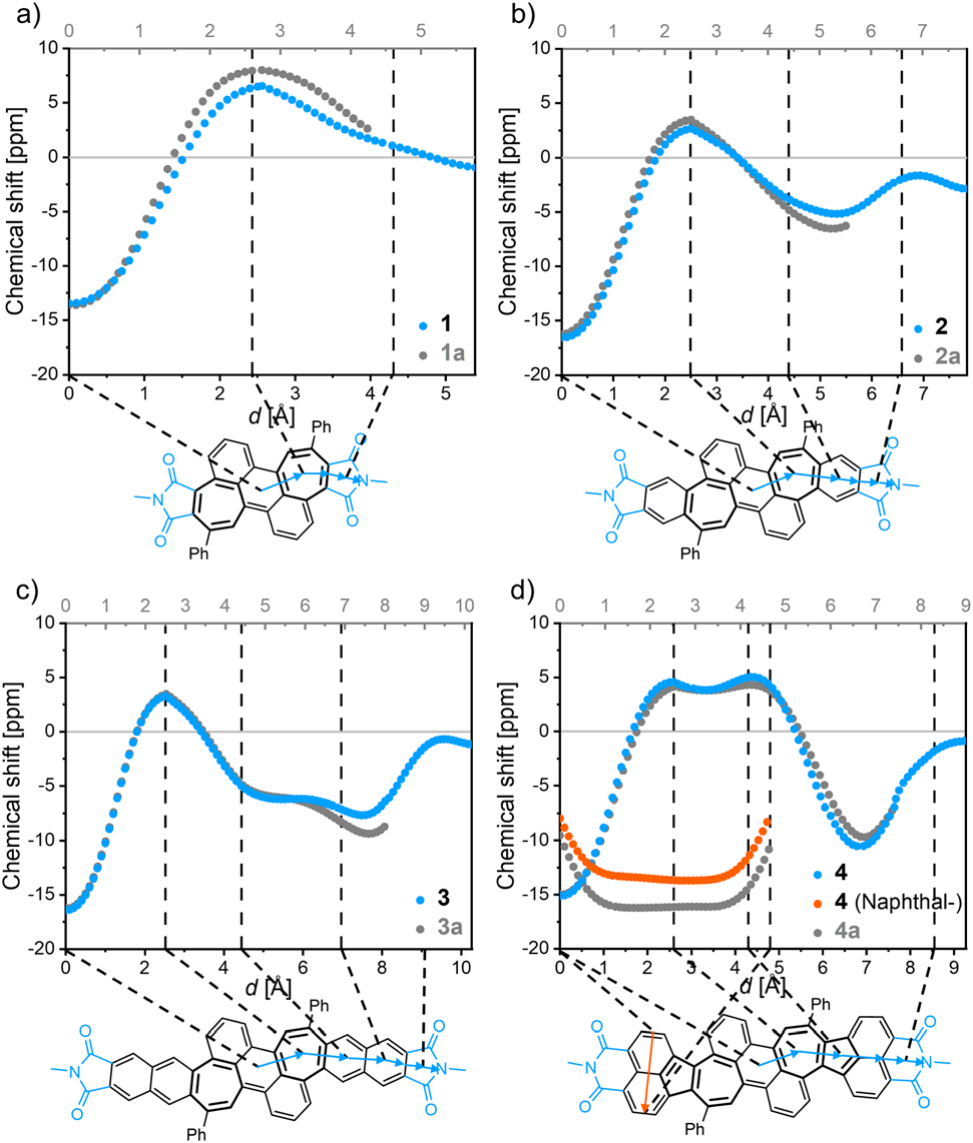
NICS-XY-scan of bisimide 1 (a), bisimide 2 (b), bisimide 3 (c), and bisimide 4 (d) denoted in blue/orange as well as their non-imidized counterparts denoted in grey, respectively. The structures of non-imidized compounds 1a to 4a correspond to the structure shown in the solid black line in the respective NICS-XY-scans of 1–4. Calculations were conducted at the GIAO-B3LYP/6-311+G(d) level of theory.

All bisimides 1–4 (and their non-imidized parent compounds 1a–4a, Fig. 4 and S20†) have similar NICS_π,*zz*_ values between −13 and −17 ppm at their central ring of the anthracene subunit. Starting now at this point and following the scan trajectory towards the imide moiety, the NICS_π,*zz*_ values increase towards positive values with a maximum at the center of the heptagonal ring, attesting a local paratropic current. The π-extensions of the bisimides 2 and 3 give rise to moderately strong diatropic currents in form of a local benzenoid and a semiglobal naphthalenoid, respectively. Acenaphthylimide 4 showed positive NICS_π,*zz*_ values for the heptagonal as well as the pentagonal rings without a significantly pronounced minimum between the ring centers, attesting the embedded azulene moiety a semiglobal paratropic current. The pendant naphthalene unit of 4 shows a strong semiglobal diatropic ring current, which was verified by an additional NICS-XY scan (Fig. 4, orange trajectory).

Comparison of the NICS-XY-scans of the shared core structure of the bisimides 1–4 and their non-imidized parent molecules 1a–4a revealed the impact of imide moieties on aromaticity (Fig. 4). The most distinct difference between the imidized and non-imidized parent compounds can be seen in the respective rings directly adjacent to the imide groups. All investigated molecules showed lower absolute NICS_π,*zz*_ values upon imidization at these rings. The NICS_π,*zz*_ values of imidized molecules 1–4 merge into equal values as those of corresponding non-imidized molecules 1a–4a for all other more distant rings. The imide rings possess nearly zero NICS_π,*zz*_ value with the same sign as the neighboring rings (that is for 1 a positive value and for 2–4 a negative value), which indicates that the apparent ring current of the imide groups originates from their direct neighbor. The calculated NICS_π,*zz*_ values for the imide moieties of 1–4 suggest more localization of π-electrons for 1 with a positive value than for 2–4 with negative values, which could further be verified by bond length alternations calculated for these imide rings (ESI, Table S5†). The results obtained by NICS_π,*zz*_ calculations were corroborated by using the ACID method, which is an intuitive and straightforward tool due to its graphical visualization (ESI, Fig. S21†).^40,41^ The results obtained by ACID agree in all respects with those of the NICS-XY-scan.

### Electrochemical measurements

Electrochemical properties of the synthesized bisimides were investigated by cyclic voltammetry (CV) and differential pulse voltammetry (DPV) using the ferrocene/ferrocenium (Fc/Fc^+^) couple as reference (Table 1). CV traces of bisimide 1 showed a reduction potential at −1.35 V, which is similar to that observed for its alternant analogue, perylene-2,3:8,9-bis(dicarboximide) (PBI) (−1.28 V).^42^ On the contrary, the first oxidation process of 1 was observed at 0.43 V, significantly lower than that of the alternant analogue (PBI) (>1.0 V).^42^ These observations reveal the influence of an imide moiety, causing high electron affinity, and a cycloheptadienyl moiety, making oxidation easier. On the other hand, phthal- and naphthalimide derivatives 2 and 3 showed lower reduction potentials (−1.64 and −1.76 V) and higher oxidation potentials (0.59 V and 0.83 V) than those of 1, and thereby leading to wider electrochemical gaps. Acenaphthalimide 4 possesses a higher reduction potential (−1.26 V) and even lower oxidation potential (0.06 V) than those of 1. Thus, the electrochemical gaps for the four bisimides are 3 > 2 ≫ 1 > 4. These experimental results support the CT-character observed for 1 and 4. Bisimides 1 and 4 also showed irreversible/quasi-reversible oxidation and reduction processes in their CV traces. This indicates electrochemical decomposition of these bisimides, which was analogously observed in aerobic solution for bisimide 4. This azulene-embedded bisimide showed an exceptionally low oxidation potential of 0.06 V (as a comparison, highly stable tetraarylated PBIs show *E*_ox1_ ≈ 1.0 V (ref. 43)), which may be related to its instability, making it prone to oxidative decomposition under aerobic conditions (Table 1).

**Table 1:** Redox properties of bisimides 1–4^a^

Bisimide	*E*_red1_ [V]	*E*_red2_ [V]	*E*_red3_ [V]	*E*_ox1_ [V]	*E*_ox2_ [V]
1	−1.35	—	—	0.43	0.66
2	−1.64	—	—	0.59	0.89
3	−1.76	—	—	0.83	1.21
4	−1.26	−1.60	−2.01	0.06	0.23

a Oxidation and reduction potentials determined by differential pulse voltammetry in CH_2_Cl_2_ (0.1 M TBAHFP) *vs.* Fc^0/+^ at 298 K.

### Crystallography

**Fig. 5:**
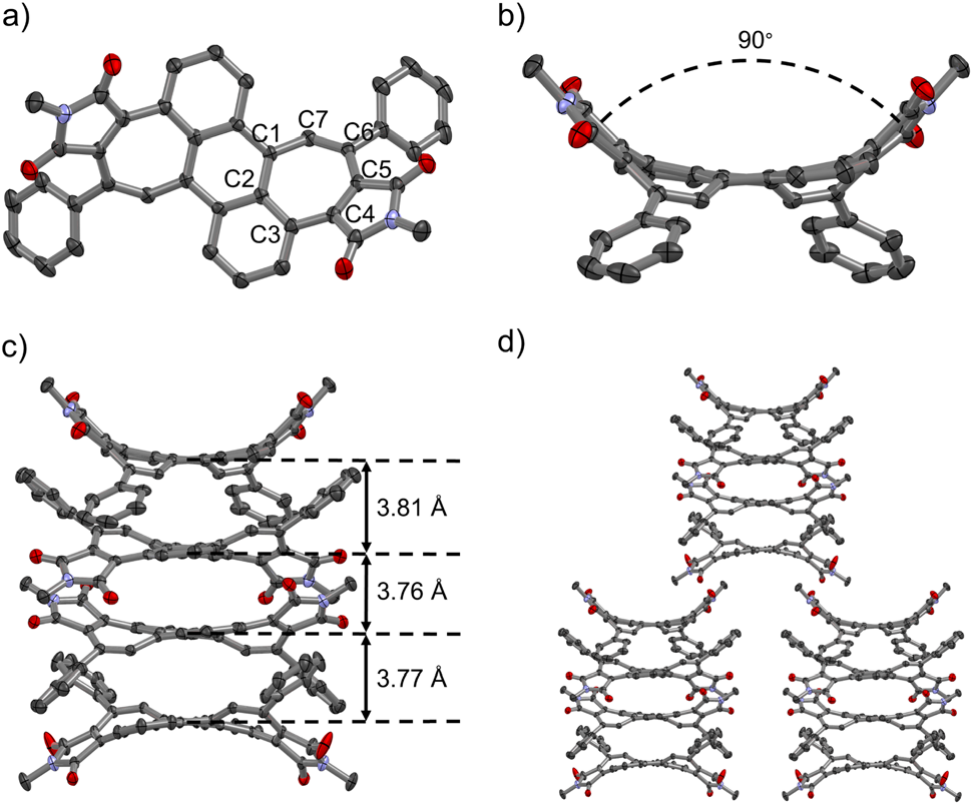
Single crystal structure of *cisoid*-shaped bisimide 1. (a) Top-down views; (b) side-on views; (c) tetralayer structure; (d) molecular arrangements in crystal packing. ORTEP drawing at 50% probability for thermal ellipsoids, hydrogen atoms and solvent molecules (CHCl_3_) are omitted for clarity.

Aromatic dicarboximides are well-known for their propensity to form tightly bond aggregates^44^ and multilayer stacks.^45^ Indeed, despite the pronounced contortion out of planarity, we could observe tetralayer stacks in the crystal structure of bisimide 1. Single crystals of 1 suitable for X-ray diffraction could be grown by slow evaporation of CHCl_3_/hexane solutions (Fig. 5). Bisimide 1 packs in tetramers with weak π–π interactions at a distance of *ca.* 3.8 Å between the central hexagonal rings (Fig. 5c). One tetramer is neighbored by four tetrameric units forming a superordinate lamellar structure of bricklayer-type due to C–H–π interactions between adjacent tetramers (Fig. 5d). Each molecule of 1 shows a highly curved saddle-shaped structure where both imide groups are located on the same side with regard to the central anthracene plane (therefore named here as *cisoid* conformation) (Fig. 5). This observation is in accordance with the DFT calculations where the *transoid* conformation is 12 kJ mol^−1^ higher in energy than the *cisoid* conformation. Evaluation of the bond lengths of the curvature inducing heptagonal moiety revealed a pronounced bond length alternation. Whereas the bond lengths of C1–C2, C2–C3, C3–C4, C5–C6, and C7–C1 are in the range of 1.418–1.475 Å clearly indicating antiaromatic character (C_antiar._–C_antiar._ = 1.41–1.50 Å),^46^ the bonds between C4–C5 (1.345 Å) and C6–C7 (1.354 Å) are significantly shorter and comparable to the length of a conjugated double bond (–C=C– = 1.339 Å) (Fig. 5a).^47^ Its highly warped topology is most obviously depicted as the nearly 90° angle between the adjacent pentagonal imide rings (Fig. 5b).

## Conclusion

In summary, we synthesized four non-alternant bis(dicarboximide)s containing a π-extended benzo[1,2:4,5]di[7]annulene moiety by a Pd-catalyzed [5 + 2] annulation of 3,9-diboraperylene and dibromo-substituted precursors. Bisimide 1 is the first aromatic dicarboximide with imide moieties directly fused to a heptagonal ring, which resulted in a CT-character for the S_0_ → S_1_ transition as confirmed by UV/vis spectroscopy and theoretical calculations by (TD-)DFT. Aromaticity studies by NICS-XY-scans and ACID plots showed the influence of the imide moieties as well as the non-alternant carbon framework on the optoelectronic properties. The highly curved *cisoid* saddle structure of 1 stacks in tetrameric units in the solid state. We anticipate that the introduction of imide groups to negatively curved, non-alternant PAHs by [5 + 2] cyclization will stimulate further activities for this unexplored substance class with the prospect of obtaining interesting functional materials, as known for their conventional counterparts, the naphthalene and perylene bisimides. Our own interests are furthermore towards solid-state co-crystalline materials similar to recently reported fullerene-templated Schwarzite-type structures.^48,49^

## Data availability

The experimental procedures, analytical data and computational details supporting the findings of this study are available within the manuscript and its ESI file.† Original spectroscopic and electrochemical data has been made openly available on Zenodo at https://doi.org/10.5281/zenodo.8307995.

## Author contributions

J. S.: conceptualization, investigation, formal analysis, visualization, writing – original draft; C. Z.: conceptualization, investigation, visualization; K. S.: conceptualization, investigation (crystallography), formal analysis (crystallography), supervision, writing – review & editing; F. W.: conceptualization, supervision, writing – review & editing, founding acquisition.

## Conflicts of interest

There are no conflicts to declare.

## Supplementary Material

SC-014-D3SC04015A-s001

SC-014-D3SC04015A-s002
