# Supplementary material for: π-Extended benzo[1,2:4,5]di[7]annulene bis(dicarboximide)s – a new class of non-alternant polycyclic aromatic dicarboximides
Source: Chem Sci. 2023 Sep 20;14(39):10861–6. doi: 10.1039/d3sc04015a (PMC10566470; doi:10.1039/d3sc04015a)
Supplement: SC-014-D3SC04015A-s001 [file SC-014-D3SC04015A-s001.pdf]

Electronic Supplementary Material (ESI) for Chemical Science.
This journal is © The Royal Society of Chemistry 2023

π-Extended Benzo[1,2:4,5]di[7]annulene Bis(dicarboximide)s – A New Class of Non-alternant Polycyclic Aromatic Dicarboximides

Jonas Spengler,[‡,a] Chongwei Zhu,[‡,§,a] Kazutaka Shoyama,[a] and Frank Würthner[a]*

[a.] *Universität Würzburg, Institut für Organische Chemie and Center for Nanosystems Chemistry, Am Hubland, 97074 Würzburg, Germany*

[‡]*These authors contributed equally to this work.*

[§]*Current address: Laboratory of Functional Molecular Solids, Ministry of Education, and School of Chemistry and Materials Science Anhui Normal University, Wuhu 241002, P. R. China.*

Supplementary Information

Table of Contents

1 Materials and Methods 2

2 Experimental Procedures 4

3 Spectra 10

3.1 NMR Spectra 10

3.2 Cyclic Voltammetry 17

3.3 UV/Vis Spectra 18

4 Crystal Structure Data 19

5 Computational Data 20

5.1 Frontier Orbitals 20

5.2 Natural Transition Orbitals 21

5.3 Overlap Integrals S_{HL} 21

5.4 TD-DFT Calculations 22

5.5 NICS Data 24

5.6 NICS(1.7)-*XY*-Scan 25

5.7 ACID Plots 27

5.8 Bond Lengths 28

5.9 Optimized Structures 29

6 References 35

1 Materials and Methods

General remarks: The chemicals were purchased from commercial suppliers and were used without further purification. Flash column chromatography was performed with silica-gel from Macherey-Nagel (particle size 40–63 µm) as stationary phase or on an interchim PuriFlash 420 system with prepacked PF-30SIHPFOO25 columns (silica-gel). The identification *via* thin layer chromatography (TLC) was performed on aluminum plates coated with 0.20 mm silica-gel containing a fluorescent indicator (Macherey-Nagel, ALUGRAM®, SIL G/UV_{254}). Dry methylene chloride was obtained from a solvent purification system PS-M6-6/7 from inert technologies. All reactions involving oxygen- or moisture-sensitive compounds were performed by standard Schlenk technique under an inert atmosphere of nitrogen. 1,3-Diisopropylimidazol-2-ylidene borane was prepared by an adapted literature method.[1]

UV/Vis absorption spectroscopy was measured on a Jasco V-670 spectrophotometer with 1 cm Hellma quartz glass cuvettes.

NMR spectroscopy was measured on Bruker Avance III HD 400 spectrometers at 298 K with deuterated methylene chloride as solvent. Chemical shifts are reported in δ units relative to tetramethylsilane and calibrated to the residual solvent signal ($CHDCl_2$ in CD_2Cl_2 at δ = 5.32 ppm).[2] Multiplicity is designated by the following abbreviations: s (singlet), d (doublet), dd (doublet of doublets) or m (multiplet). Coupling constants are reported as observed and given in Hz. All NMR spectra were analyzed and processed with MestReNova v14.2.1.

Mass spectra were measured on a Bruker Daltonics ultrafleXtreme mass spectrometer (matrix-assisted laser desorption/ionisation time-of-flight, MALDI-TOF) using *trans*-2-[3-(4-*tert*-butylphenyl)-2-methyl-2-propenylidene]malononitrile (DCTB) as the matrix.

Cyclic and differential pulse voltammetry

Cyclic voltammetry (CV) and differential pulse voltammetry (DPV) measurements were carried out with a *BASi* Epsilon potentiostat connected to a microcell apparatus from *rhd instruments* involving a 1.6 mL sample container, a platinum counter- and pseudo-reference electrode as well as a glassy carbon working electrode. Tetrabutylammonium hexafluorophosphate ($(n\text{-}Bu)_4NPF_6$) was applied as the

supporting electrolyte with ferrocene (Fc) as an internal standard for the calibration of potentials.

Single crystal X-ray analysis was carried out on Bruker D8 Quest Kappa diffractometers with a PhotonII CMOS detector and multilayered mirror monochromated CuK_{α} radiation (λ = 1.54178 Å). The structure was solved using SHELXT[3] expanded with Fourier techniques and refined using the SHELX software package.[4] Hydrogen atoms were assigned at idealized positions and were included in the calculation of structure factors. All non-hydrogen atoms in the main residue were refined anisotropically. Standard SHELX restraints RIGU, DELU, ISOR, SAME, and SIMU were applied to model disordered chloroform solvates.

Melting points were determined with a Stuart SMP50 automatic digital melting point apparatus or a polarization microscope BX41 from Olympus in conjunction with a temperature control element TP-94 by Linkam. All given melting points are uncorrected.

Theoretical calculations: Geometry optimizations, $NICS(1)_{zz}$ values[5], and AICD[6] calculations were performed using density functional theory (DFT) as implemented in the Gaussian 16 program (Revision A.03) at the B3LYP/6-31+G(d) level of theory.[7] $NICS_{\pi,zz}$-*XY*-Scans were calculated at the GIAO-B3LYP/6-311+G(d) level of theory using the Aroma 1.0 utility package developed by Stanger and coworkers[8–11] as a “Plug-In” utility for Gaussian 09 (Revision A.02).[12] The AICD software package for visualization was provided by Prof. R. Herges.[6] TD-DFT calculations were performed at the CAM-B3LYP/6-31+G(d) level of theory. The overlap degree S_{HL} between HONTO and LUNTO ($\int |\varphi_H(\boldsymbol{r})|\,|\varphi_L(\boldsymbol{r})| d\boldsymbol{r}$) was calculated via multiwfn[13] using Becke's grid-based integration approach.[14] To prepare and analyze $NICS(1)_{zz}$ values for non-planar systems, the multiwfn[13] software package was used.

2 Experimental Procedures

5,6-Dibromo-2-methylisoindoline-1,3-dione (7).

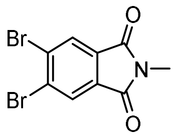

A round-bottom flask was charged with 5,6-dibromoisoindoline-1,3-dione (100 mg, 328 µmol, 1.00 equiv.), potassium carbonate (90.7 mg, 656 µmol, 2.00 equiv.), dimethylformamide (DMF) (14 mL), and methyl iodide (93.1 mg, 40.8 µL, 656 µmol, 2.00 equiv.). The resulting reaction mixture was stirred at 40 °C for 24 h, diluted with EtOAc (50 mL), filtered through Celite and concentrated under reduced pressure. Colum chromatography (silica-gel, CH_2Cl_2) with subsequent recrystallization by slow evaporation of a saturated CH_2Cl_2 solution layered with MeOH gave desired compound **7** (91 mg, 87%) as colorless needles.

^{1}H NMR (400 MHz, CD_2Cl_2): δ = 8.08 (s, 2H), 3.14 (s, 3H) ppm.

^{13}C NMR (101 MHz, CD_2Cl_2): δ = 166.8, 132.5, 131.4, 128.6, 24.5 ppm.

DIP-APCI-HRMS calcd. for $C_9H_5{}^{79}Br^{81}BrNO_2$ $[M+H]^+$: 319.8740; found: 319.8723

M.p.: >300 °C.

6,7-Dibromo-2-methyl-*1H*-benzo[*f*]isoindole-1,3(*2H*)-dione (8).

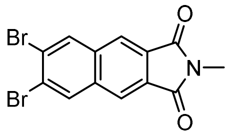

This compound was synthesized by using a modified literature procedure.[15] A round-bottom flask was charged with 1,2-dibromo-4,5-bis(dibromomethyl)benzene (950 mg, 1.64 mmol, 1.00 equiv.), *N*-methyl maleimide (248 mg, 2.23 mmol, 1.36 eq.), triethylammonium iodine (3.37 g, 13.1 mmol, 8.00 equiv.) and DMF (14 mL). The suspension was stirred at 110 °C for 16 h and treated with sat. aq. $NaHSO_3$ (30 mL). The combined organic phases were washed with water (3 × 50 mL) and concentrated under reduced pressure. Flash column chromatography (SiO_2, CH_2Cl_2:cyclohexane 1:1) with subsequent recrystallization from hot chloroform gave desired imide **8** (167 mg, 28%) as a colorless crystalline solid.

^{1}H NMR (400 MHz, CD_2Cl_2): δ = 8.39 (s, 2H), 8.23 (s, 2H), 3.21 (s, 3H) ppm.

^{13}C NMR (101 MHz, CD_2Cl_2): δ = 135.2, 134.7, 129.7, 126.3, 123.6, 24.5 ppm. (one signal superimposed to another)

DIP-APCI-HRMS calcd. for $C_{13}H_7{}^{79}Br^{81}BrNO_2$ $[M+H]^+$: 369.8896; found: 369.8889.
M.p.: 209–210 °C.

6,7-Dibromo-2-methyl-*1H*-indeno[6,7,1-*def*]isoquinoline-1,3(*2H*)-dione (9).

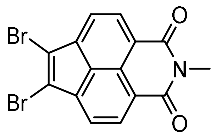

In a round bottom flask with condenser 2-methyl-6,7-dihydro-*1H*-indeno[6,7,1-*def*]isoquinoline-1,3(*2H*)-dione (1.00 g, 4.21 mmol, 1.00 equiv.), *N*-bromosuccinimide (5.22 g, 29.5 mmol, 7.00 equiv.), and benzoyl peroxide (102 mg, 421 µmol, 0.100 equiv.) were suspended in chlorobenzene (90 mL). The mixture was stirred at 137 °C for 19 h and subsequently treated with sat. aq. $NaHCO_3$ (100 mL). The combined organic phases were washed with water (3 × 100 mL) and dried over Na_2SO_4. After evaporation of the solvent, recrystallisation form hot chloroform gave desired compound **9** (786 mg, 47%) as a crystalline red solid.

^1H NMR (400 MHz, CD_2Cl_2): δ = 8.33 (d, *J* = 7.1 Hz, 2H), 7.72 (d, *J* = 7.0 Hz, 2H), 3.48 (s, 3H) ppm.
^{13}C NMR (101 MHz, CD_2Cl_2): δ = 163.8, 141.6, 131.8, 126.2, 124.9, 124.3, 123.8, 122.8, 27.2 ppm.
MALDI-HRMS (DCTB) calcd. for $C_{15}H_7{}^{79}Br_2NO_2$ $[M]^+$: 390.8838; found: 390.8865
M.p.: 270–272 °C (decomp.).

***N,N'*-Dimethyl-6,13-diphenyl-dicyclohepta[1,2,3-*de*:1',2',3'-*kl*]anthracene 4,5:11,12-bis(dicarboximide) (1).**

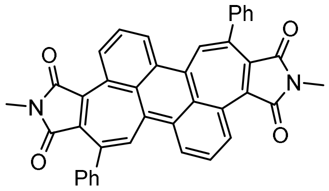

Under nitrogen atmosphere, a Schlenk tube was charged with 2,8-diphenylanthra[1,9-*bc*:5,10-*b'c'*]bis(borinine)-3,9-diol (**5**) (25.0 mg, 57.6 µmol, 1.00 equiv.), 3,4-dibromo-1-methyl-*1H*-pyrrole-2,5-dione (**6**) (46.5 mg, 173 µmol, 3.00 equiv.), bis-(dibenzylidene-acetone)-palladium(0)•chloroform (7.15 mg, 6.91 µmol, 12.0 mol %), tri-*tert*-butyl-phosphonium tetrafluoroborate (4.68 mg, 16.1 µmol, 28.0 mol %), caesium carbonate (124 mg, 380 µmol, 6.60 equiv.), water (30 µL, 29.9 µg, 1.66 µmol, 2.88 mol %), and

tert-amyl alcohol (9.5 mL). The resulting suspension was stirred at r.t. for 1 h and subsequently heated to 100 °C for 62 h. After the mixture was filtered through Celite®, the solvents were evaporated, and the crude mixture subjected to column chromatography (silica-gel, CH_2Cl_2). Further purification by HPLC (silica-gel, CH_2Cl_2) gave desired bisimide **1** (5.8 mg, 17%) as a dark red solid.

^{1}H NMR (400 MHz, CD_2Cl_2): δ = 8.13 (d, *J* = 7.7 Hz, 2H), 7.83 (d, *J* = 7.3 Hz, 2H), 7.59 (dd, *J* = 8.7, 7.3 Hz, 2H), 7.49–7.42 (m, 10H), 7.02 (s, 2H), 3.02 (s, 6H) ppm.
^{13}C NMR (101 MHz, CD_2Cl_2): δ = 169.6, 168.4, 141.4, 141.0, 140.4, 140.0, 137.7, 136.6, 132.1, 129.8, 129.7, 128.9, 128.5, 128.1, 127.4, 127.3, 125.2, 24.5 ppm.
UV/Vis (CH_2Cl_2): $\lambda_{max}(\varepsilon)$ = 293 (31600), 518 (9100) nm.
MALDI-HRMS (DCTB) calcd. for $C_{40}H_{24}N_2O_4$ $[M]^+$: 596.1736, found: 596.1730.
Cyclic voltammetry (CH_2Cl_2, 0.1 M (n-Bu)$_4$NPF$_6$, against $Fc^{0/+}$): E_{red}^1 = −1.35 V, E_{ox}^1 = +0.43 V; E_{ox}^2 = +0.66 V.
M.p.: 167–168 °C.

***N*,*N'*-Dimethyl-8,17-diphenyl-bis(benzo[6,7]cyclohepta)[1,2,3-*de*:1',2',3'-*kl*]anthracene 5,6:14,15-bis(dicarboximide) (2).**

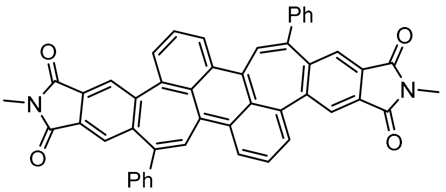

Under nitrogen atmosphere, a Schlenk tube was charged with 2,8-diphenylanthra[1,9-*bc*:5,10-*b'c'*]bis(borinine)-3,9-diol (**5**) (20.0 mg, 46.1 µmol, 1.00 equiv.), 5,6-dibromo-2-methylisoindoline-1,3-dione (**7**), (44.1 mg, 138 µmol, 3.00 equiv.) bis-(dibenzylidene-acetone)-palladium(0) chloroform adduct (4.77 mg, 4.61 µmol, 10.0 mol %), tri-*tert*-butylphosphonium tetrafluoroborate (3.21 mg, 11.1 µmol, 24.0 mol %), caesium carbonate (99.0 mg, 304 µmol, 6.60 equiv.), water (20 µL, 19.9 µg, 1.11 µmol, 2.41 mol %) and *tert*-amyl alcohol (10 mL). The resulting suspension was stirred at r.t. for 1 h and subsequently stirred at 100 °C for 65 h. After cooled down to room temperature, the mixture was filtered through Celite® and concentrated under reduced pressure. The crude mixture was subjected to column chromatography (silica-gel,

CH_2Cl_2:MeOH 100:1; hexane:EtOAc 3:1). The desired bisimide **2** (4.1 mg, 15%) was obtained as a dark red solid.

^{1}H NMR (400 MHz, CD_2Cl_2): δ = 8.34 (dd, *J* = 8.3, 1.5 Hz, 2H), 7.74–7.67 (m, 4H), 7.62–7.59 (m, 4H), 7.54 (s, 2H), 7.52–7.40 (m, 10 H), 3.10 (s, 6H) ppm.
^{13}C NMR (101 MHz, CD_2Cl_2): δ = 168.2, 148.2, 145.0, 143.4, 142.6, 138.1, 137.0, 135.2, 133.1, 131.1, 130.7, 130.6, 129.3, 129.2, 129.1, 128.8, 128.7, 128.5, 128.2, 124.7, 124.4, 24.2 ppm.
UV/Vis (CH_2Cl_2): $\lambda_{max}(\varepsilon)$ = 292 (50600), 519 (20000) nm.
MALDI-HRMS (DCTB) calcd. for $C_{48}H_{28}N_2O_4$ $[M]^+$: 696.2044; found: 696.2054.
E_{red}^1 = −1.64 V, E_{ox}^1 = +0.59 V; E_{ox}^2 = +0.89 V.
M.p.: 229–231 °C.

***N,N'*-Dimethyl-10,21-diphenyl-bis(naphtho[2'',3'':6,7]cyclohepta)[1,2,3-*de*:1',2',3'-*kl*]anthracene 6,7:17,18-bis(dicarboximide) (3).**

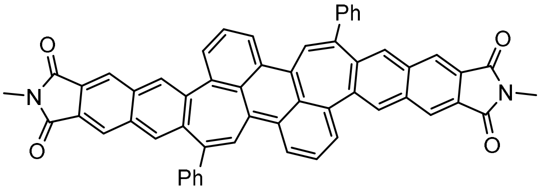

Under nitrogen atmosphere, a Schlenk tube was charged with 2,8-diphenylanthra[1,9-*bc*:5,10-*b'c'*]bis(borinine)-3,9-diol (**5**) (20.0 mg, 46.1 µmol, 1.00 equiv.), dibromo imide **8** (51.0 mg, 138 µmol, 3.00 equiv.), bis-(dibenzylideneacetone)-palladium(0) chloroform adduct (4.77 mg, 4.61 µmol, 10.0 mol %), tri-*tert*-butylphosphonium tetrafluoroborate (3.11 mg, 10.7 µmol, 23.0 mol %), caesium carbonate (99.0 mg, 304 µmol, 6.60 equiv.), water (30 µL, 29.9 µg, 1.66 µmol, 3.60 mol %), and *tert*-amyl alcohol (9.5 mL). The resulting suspension was stirred at r.t. for 1 h and subsequently stirred at 100 °C for 65 h. The mixture was cooled down to room temperature, filtered through Celite®, and concentrated under reduced pressure. The crude mixture subjected to column chromatography (silica-gel, CH_2Cl_2:MeOH 200:1), and subsequently purified by HPLC (silica-gel, CH_2Cl_2:MeOH 500:3) to give desired bisimide **3** (1.9 mg, 5%) as a dark red solid.

^{1}H NMR (600 MHz, CD_2Cl_2): δ = 8.42 (dd, *J* = 8.7 Hz, *J* = 1.1 Hz, 2H), 8.20 (s, 2H), 8.03, (s, 2H), 7.80 (dd, *J* = 7.0 Hz, *J* = 1.1 Hz, 2H), 7.76 (s, 2H), 7.74–7.73 (m, 2H),

7.70–7.68 (m, 6H), 7.54–7.51 (m, 4H), 7.49-7.46 (m, 2H), 7.44 (s, 2H), 3.17 (s, 6H) ppm.

^{13}C NMR (151 MHz, CD_2Cl_2): δ = 168.2, 168.1, 144.4, 144.2, 142.5, 140.7, 137.4, 136.7, 136.4, 135.1, 134.5, 134.2, 131.0, 130.9, 130.5, 129.6, 129.2, 129.2, 128.8, 128.5, 128.5, 128.0, 124.8, 124.6, 124.4, 24.3 ppm.

UV/Vis (CH_2Cl_2): $\lambda_{max}(\varepsilon)$ = 302 (51400), 336 (16500), 540 (15300) nm.

MALDI-HRMS (DCTB) calcd. for $C_{56}H_{32}N_2O_4$ [M]$^+$: 796.2357; found: 796.2346.

$E_{red}{}^1$ = −1.76 V, $E_{ox}{}^1$ = +0.83 V; $E_{ox}{}^2$ = +1.21 V.

M.p.: >300 °C.

***N,N'*-Dimethyl-10,21-diphenyl-bis(acenaphtho[1'',2'':6,7]cyclohepta)[1,2,3-*de*:1',2',3'-*kl*]anthracene 6,7:17,18-bis(dicarboximide) (4).**

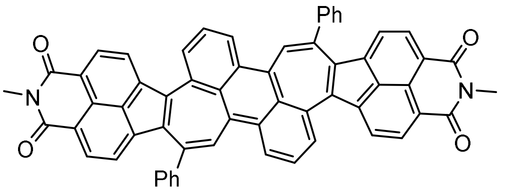

Under nitrogen atmosphere, a Schlenk tube was charged with 2,8-diphenylanthra[1,9-*bc*:5,10-*b'c'*]bis(borinine)-3,9-diol (**5**) (20.0 mg, 46.1 µmol, 1.00 equiv.), dibromo imide **9** (54.3 mg, 138 µmol, 3.00 equiv.), bis-(dibenzylideneacetone)-palladium(0) chloroform adduct (5.72 mg, 5.53 µmol, 12.0 mol %), tri-*tert*-butylphosphonium tetrafluoroborate (3.74 mg, 12.9 µmol, 28.0 mol %), caesium carbonate (99.0 mg, 304 µmol, 6.60 equiv.), water (30 µL, 29.9 µg, 1.66 µmol, 3.60 mol %), and *tert*-amyl alcohol (7.5 mL). The resulting suspension was stirred at r.t. for 1 h and subsequently stirred at 100 °C for 65 h. The mixture was cooled down to room temperature, filtered through Celite®, and concentrated under reduced pressure. The crude mixture was subjected to column chromatography (silica-gel, CH_2Cl_2:MeOH 200:1) and subsequently purified by HPLC (silica-gel, CH_2Cl_2:MeOH 200:1) and GPC to give desired bisimide **4** (1.6 mg, 4%) as a dark purple solid.

^{1}H NMR (600 MHz, CD_2Cl_2): δ = 8.27–8.23 (m, 4H), 8.03 (d, *J* = 7.4 Hz, 2H), 7.86 (d, *J* = 7.4 Hz, 2H), 7.77 (d, *J* = 7.2 Hz, 2H), 7.77–7.65 (m, 6H), 7.51–7.47 (m, 6H), 7.12 (s, 2H), 6.61 (d, *J* = 7.4 Hz, 2H), 3.44 (s, 6H) ppm.

^{13}C NMR (151 MHz, CD_2Cl_2): δ = 163.9, 163.8, 145.7, 145.0, 143.2, 142.0, 141.8, 139.6, 138.1, 135.2, 132.2, 132.0, 131.2, 131.1, 129.9, 128.8, 128.7, 128.6, 126.7,

126.5, 126.1, 124.0, 123.5, 123.4, 123.1, 122.6, 26.6 ppm. (one signal superimposed to another)

UV/Vis (CH_2Cl_2): $\lambda_{max}(\varepsilon)$ = 291 (32800), 362 (17700), 385 (21200), 406 (18700), 516 (8900) nm.

MALDI-HRMS (DCTB) calcd. for $C_{60}H_{32}N_2O_4$ $[M]^+$: 844.2357; found: 844.2336.

E_{red}^1 = −1.35 V, E_{red}^2 = −1.35 V, E_{ox}^1 = +0.43 V; E_{ox}^2 = +0.66 V.

M.p.: >300 °C.

3 Spectra

3.1 NMR Spectra

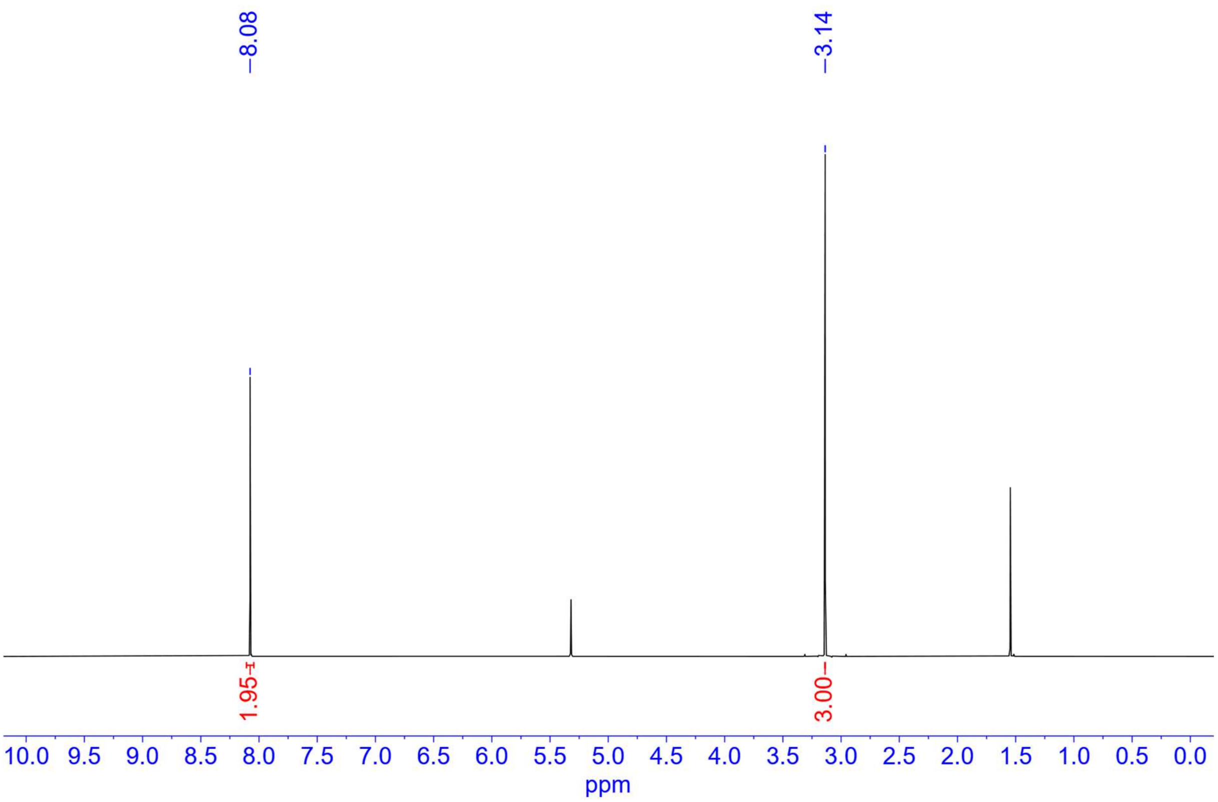

Figure S1: ^{1}H NMR (400 MHz, CD_2Cl_2) spectrum of compound **7** recorded at 298 K.

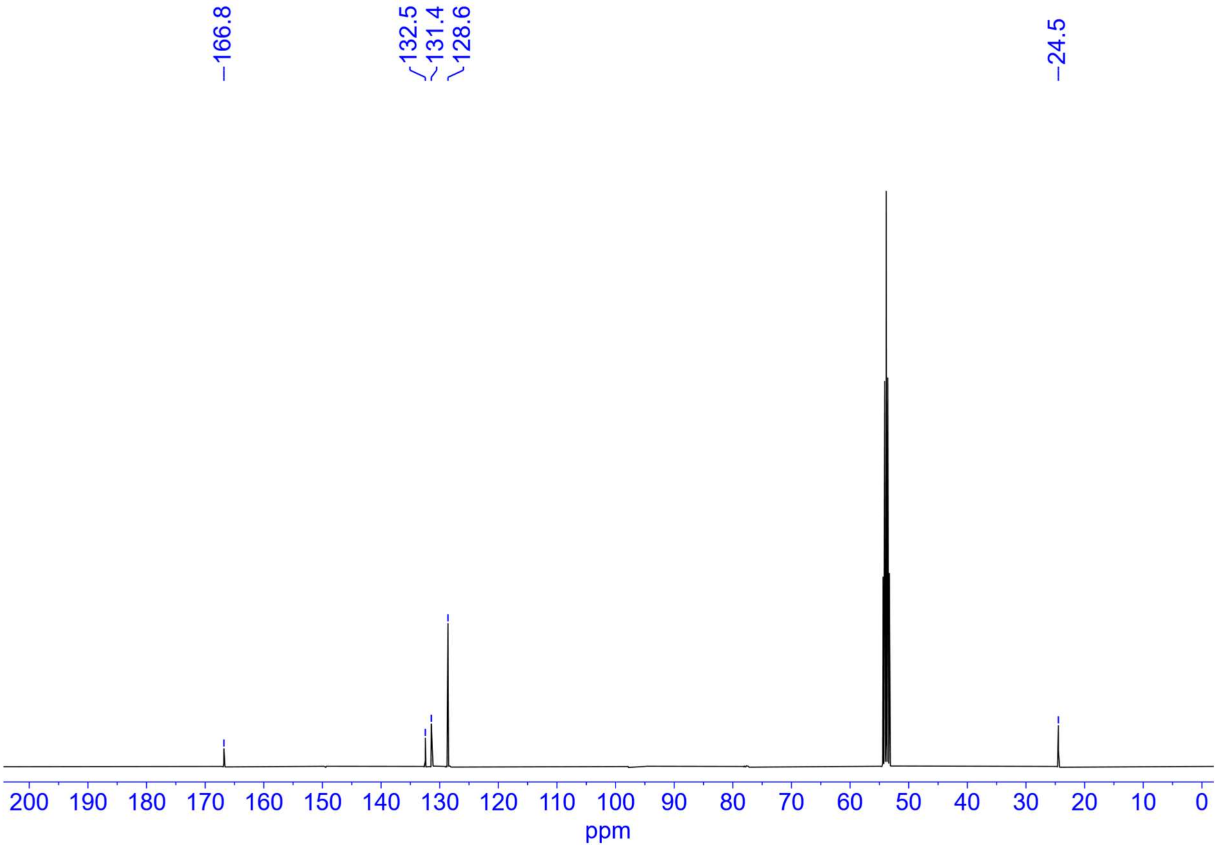

Figure S2: ^{13}C NMR (101 MHz, CD_2Cl_2) spectrum of compound **7** recorded at 298 K.

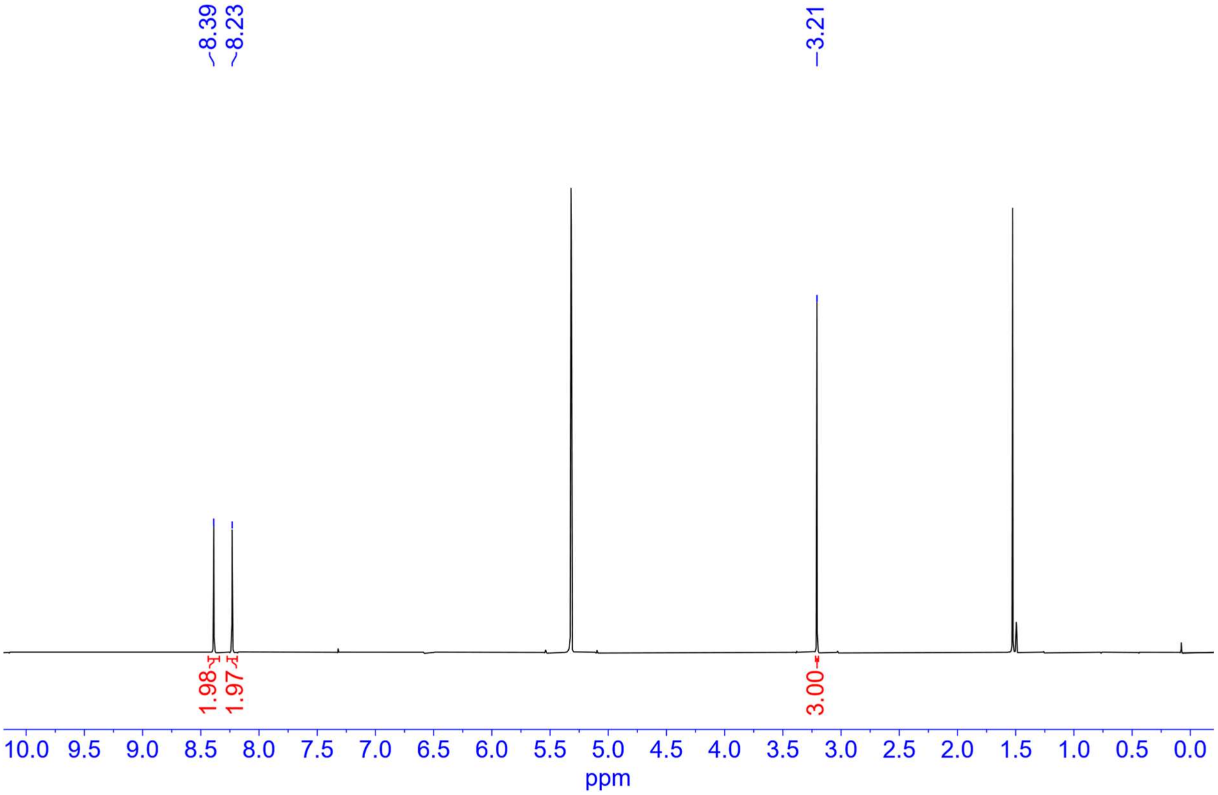

Figure S3: ^{1}H NMR (400 MHz, CD_2Cl_2) spectrum of compound **8** recorded at 298 K.

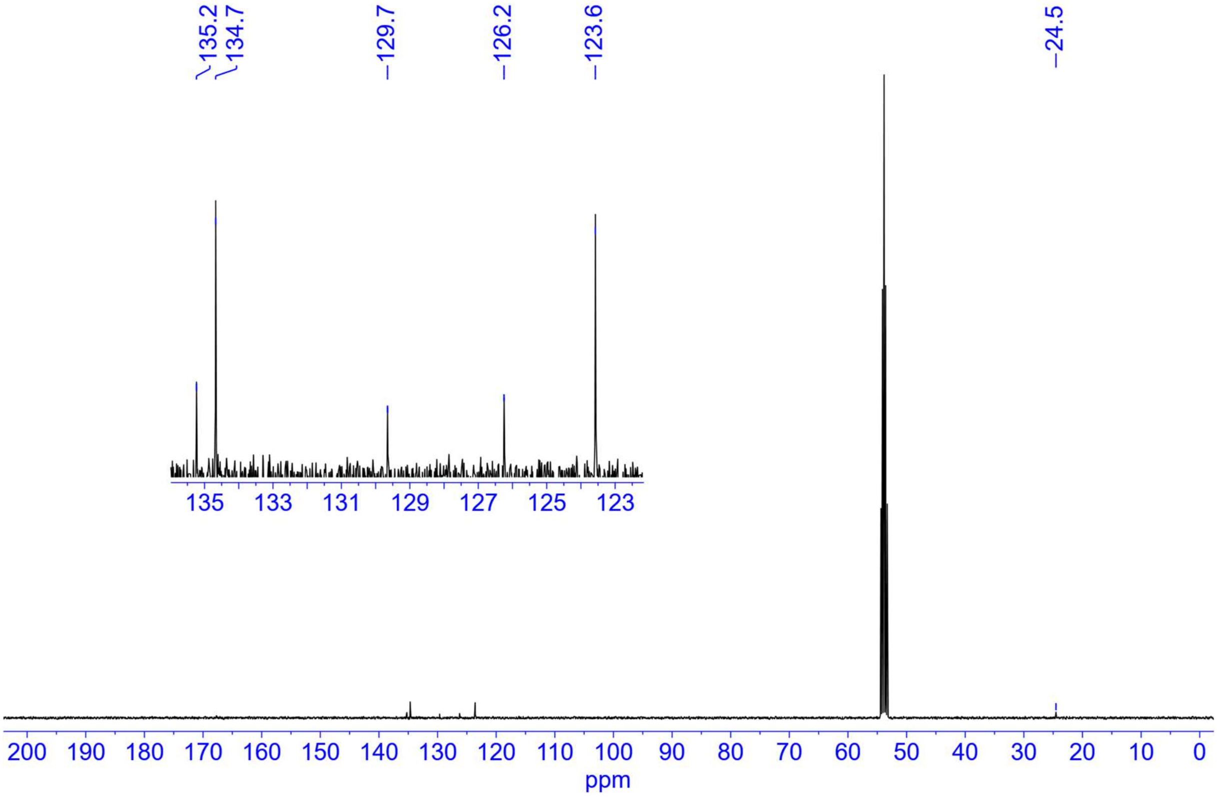

Figure S4: ^{13}C NMR (101 MHz, CD_2Cl_2) spectrum of compound **8** recorded at 298 K.

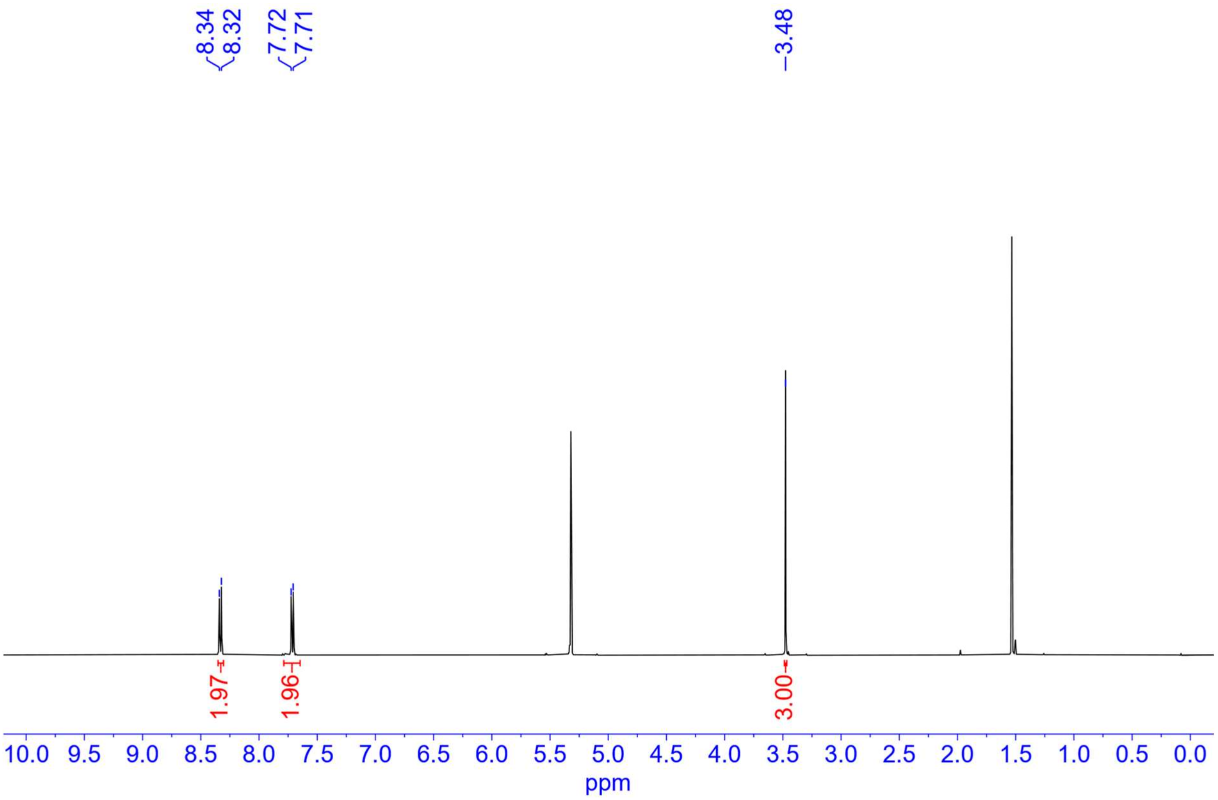

Figure S5: ^{1}H NMR (400 MHz, CD_2Cl_2) spectrum of compound **9** recorded at 298 K.

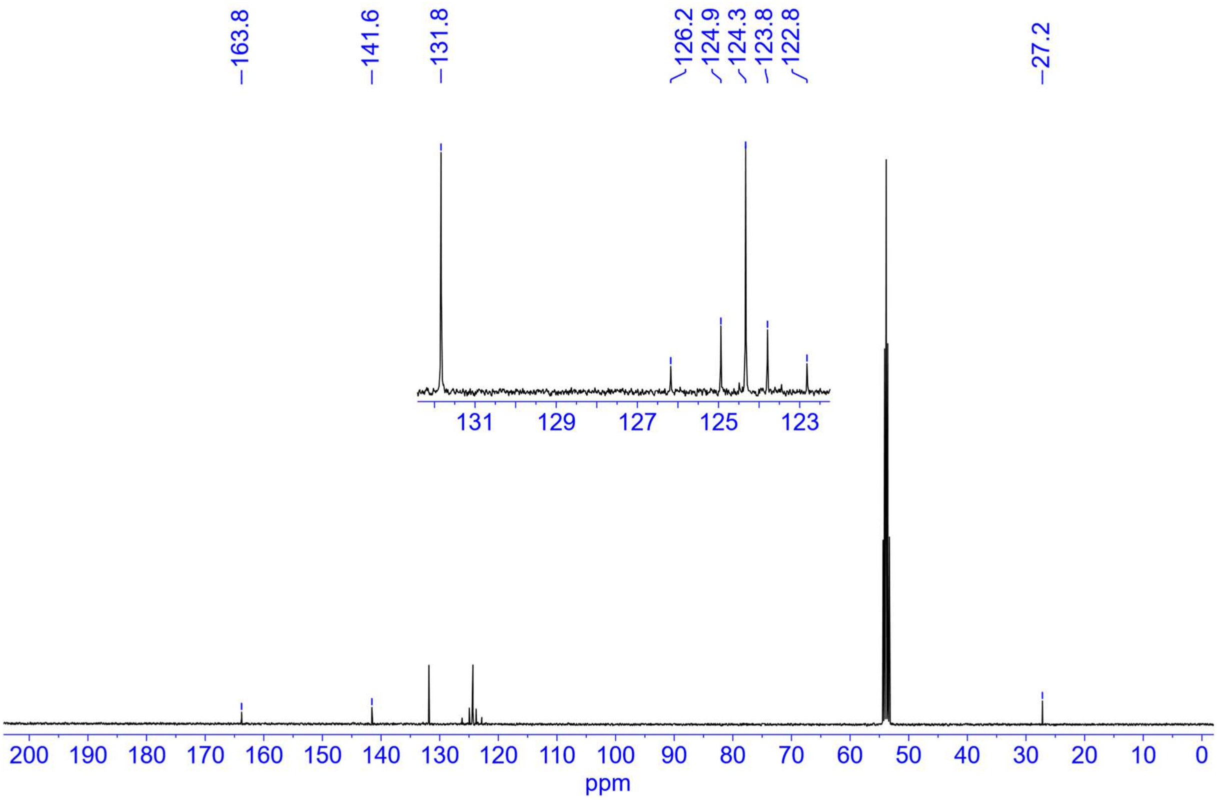

Figure S6: ^{13}C NMR (101 MHz, CD_2Cl_2) spectrum of compound **9** recorded at 298 K.

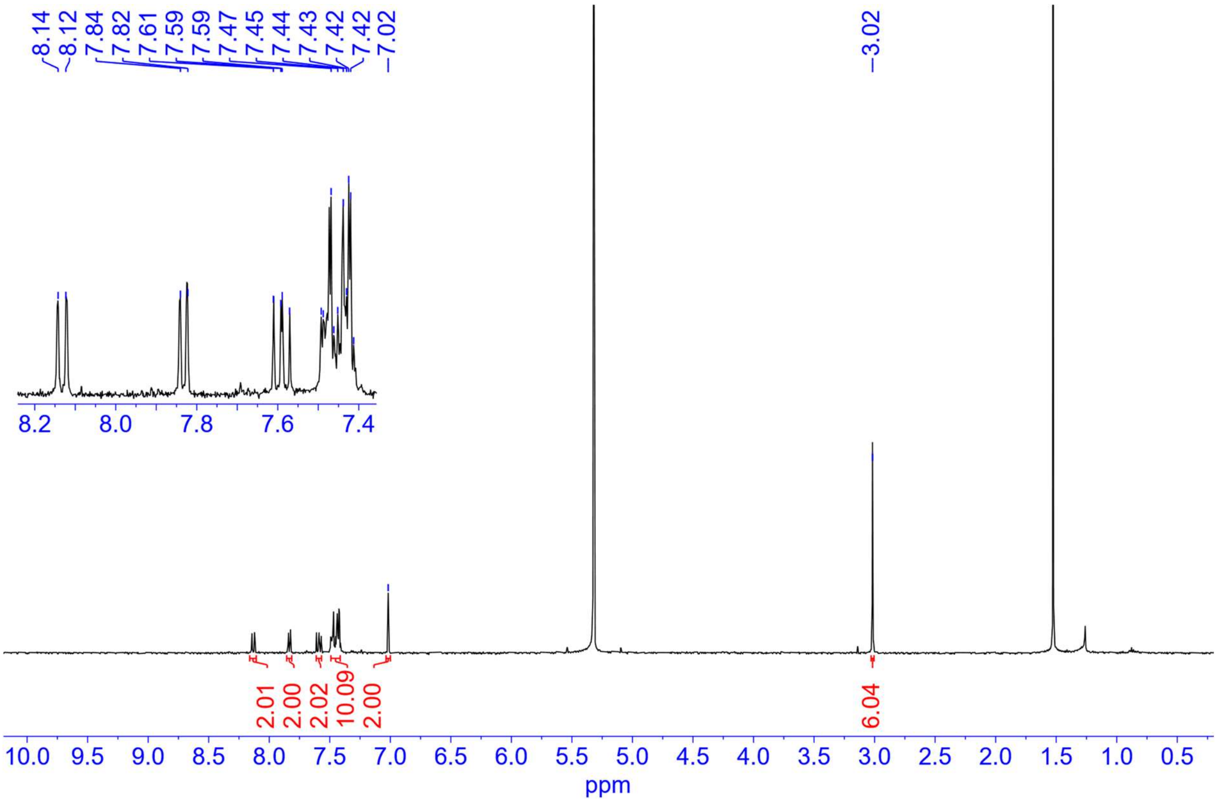

Figure S7: ^{1}H NMR (400 MHz, CD_2Cl_2) spectrum of compound **1** recorded at 298 K.

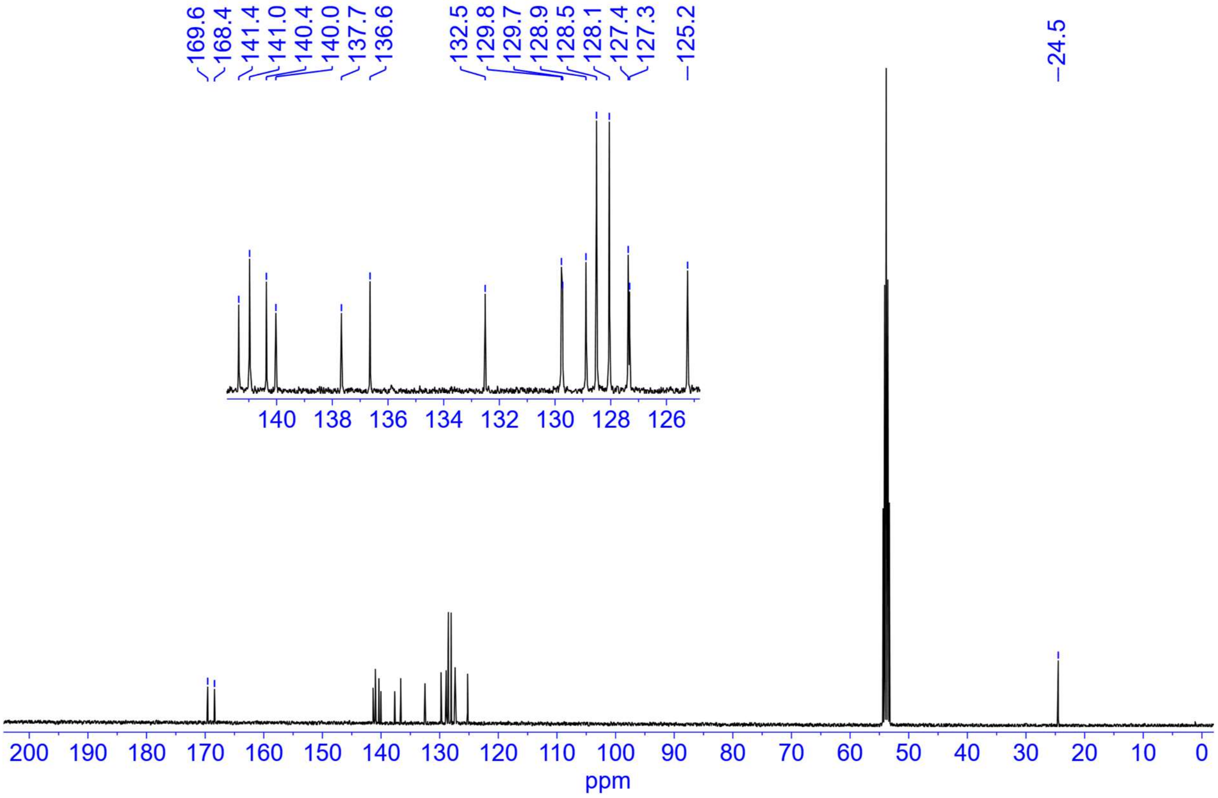

Figure S8: ^{13}C NMR (101 MHz, CD_2Cl_2) spectrum of compound **1** recorded at 298 K.

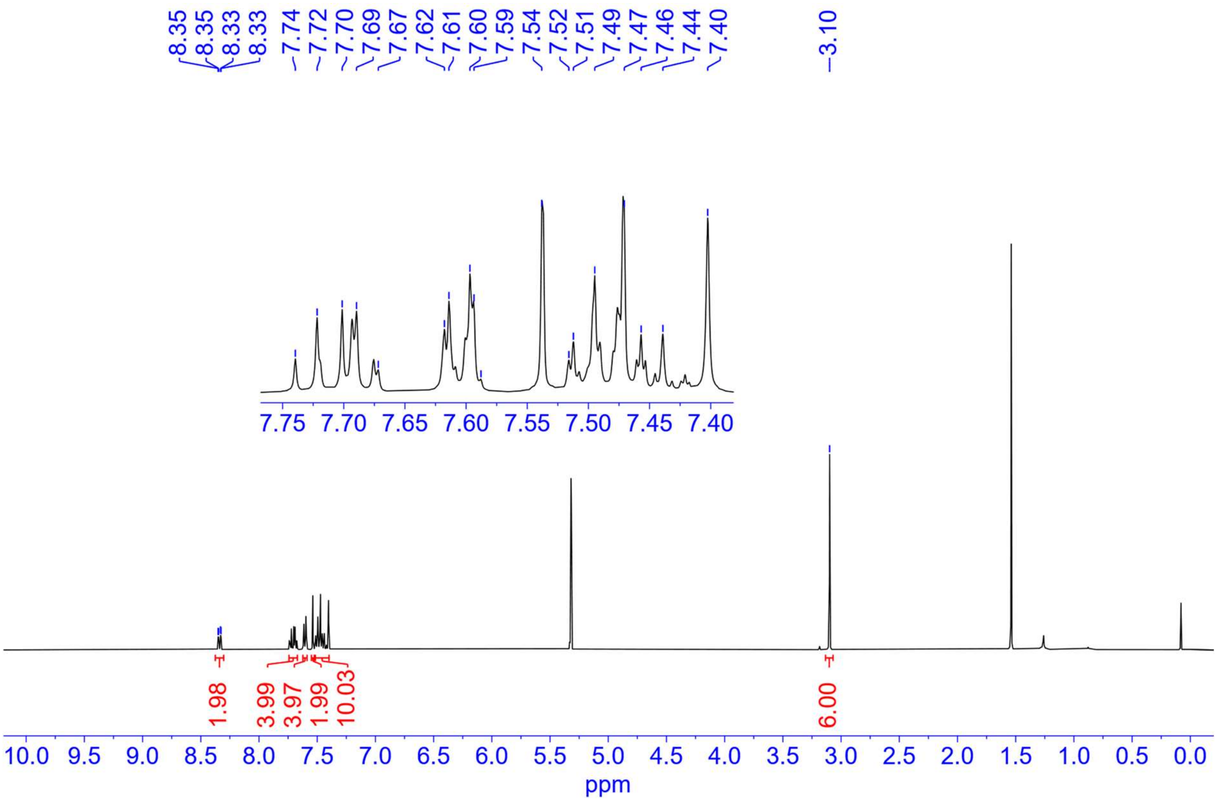

Figure S9: ^{1}H NMR (400 MHz, CD_2Cl_2) spectrum of compound **2** recorded at 298 K**.**

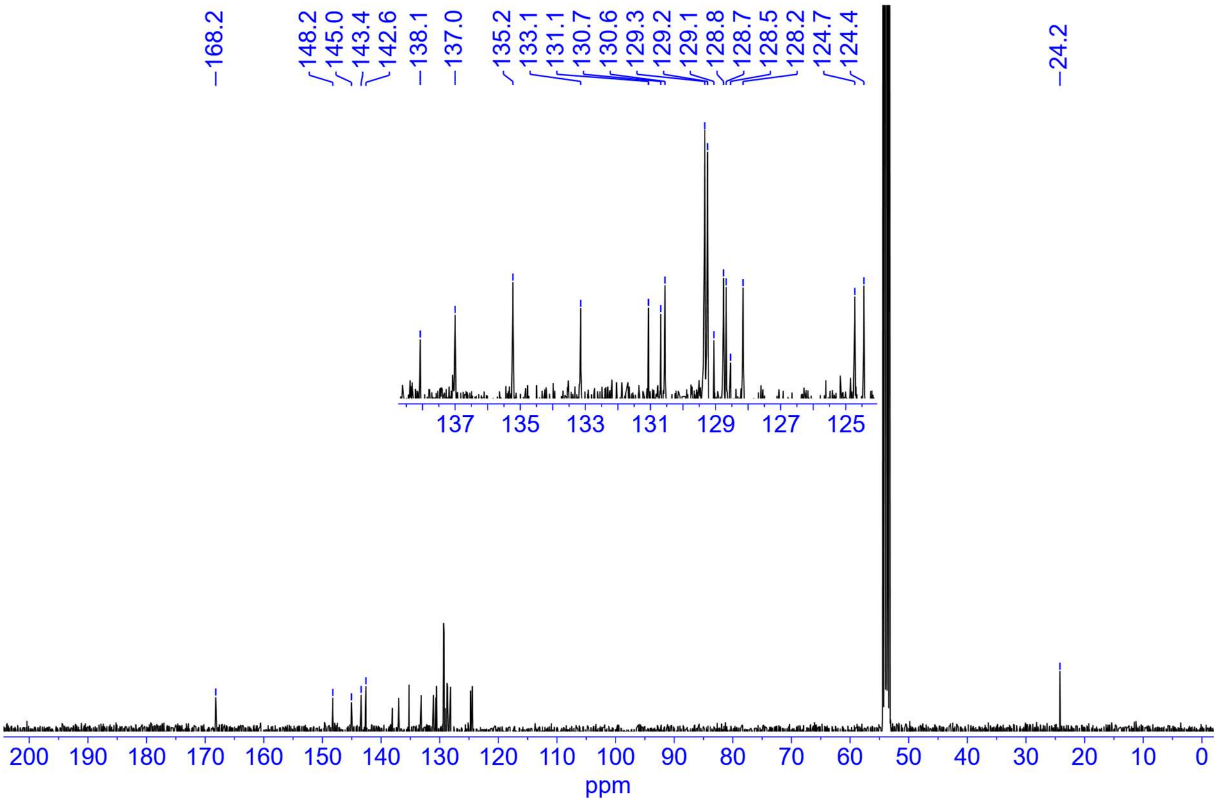

Figure S10: ^{13}C NMR (101 MHz, CD_2Cl_2) spectrum of compound **2** recorded at 298 K.

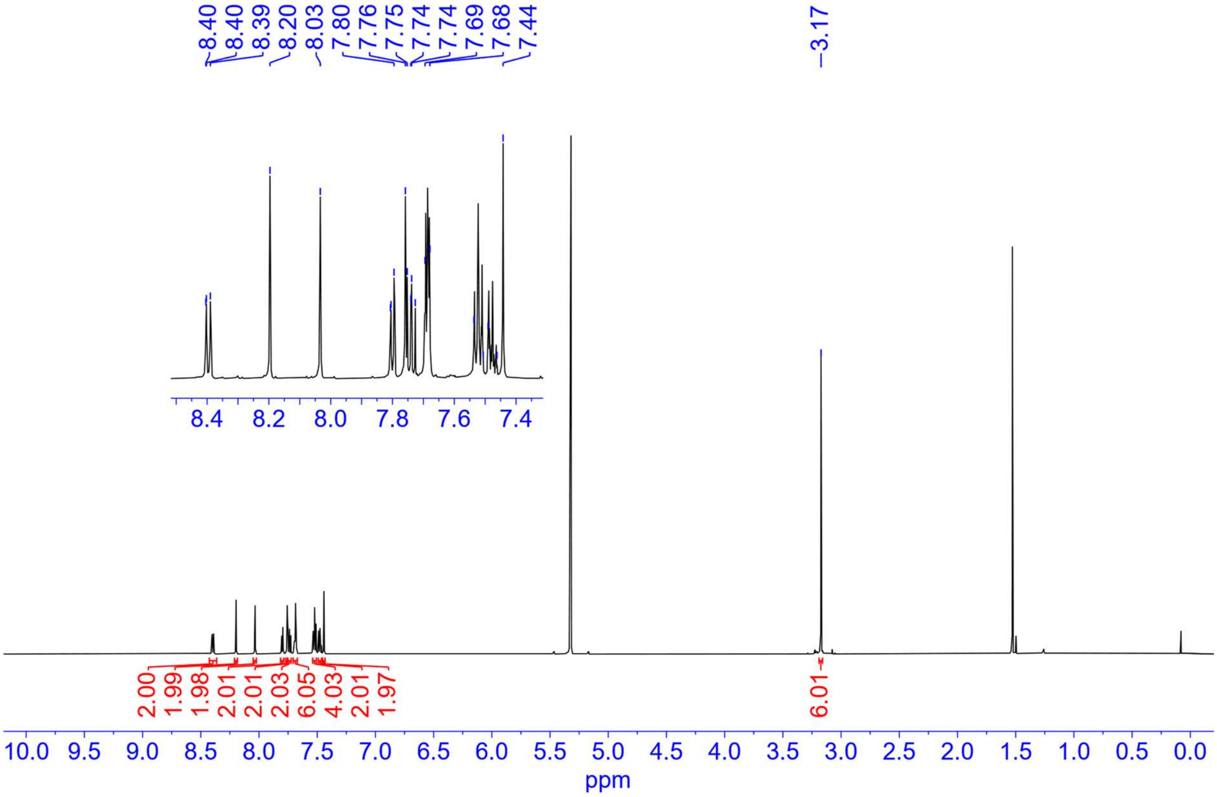

Figure S11: ^{1}H NMR (600 MHz, CD_2Cl_2) spectrum of compound **3** recorded at 298 K.

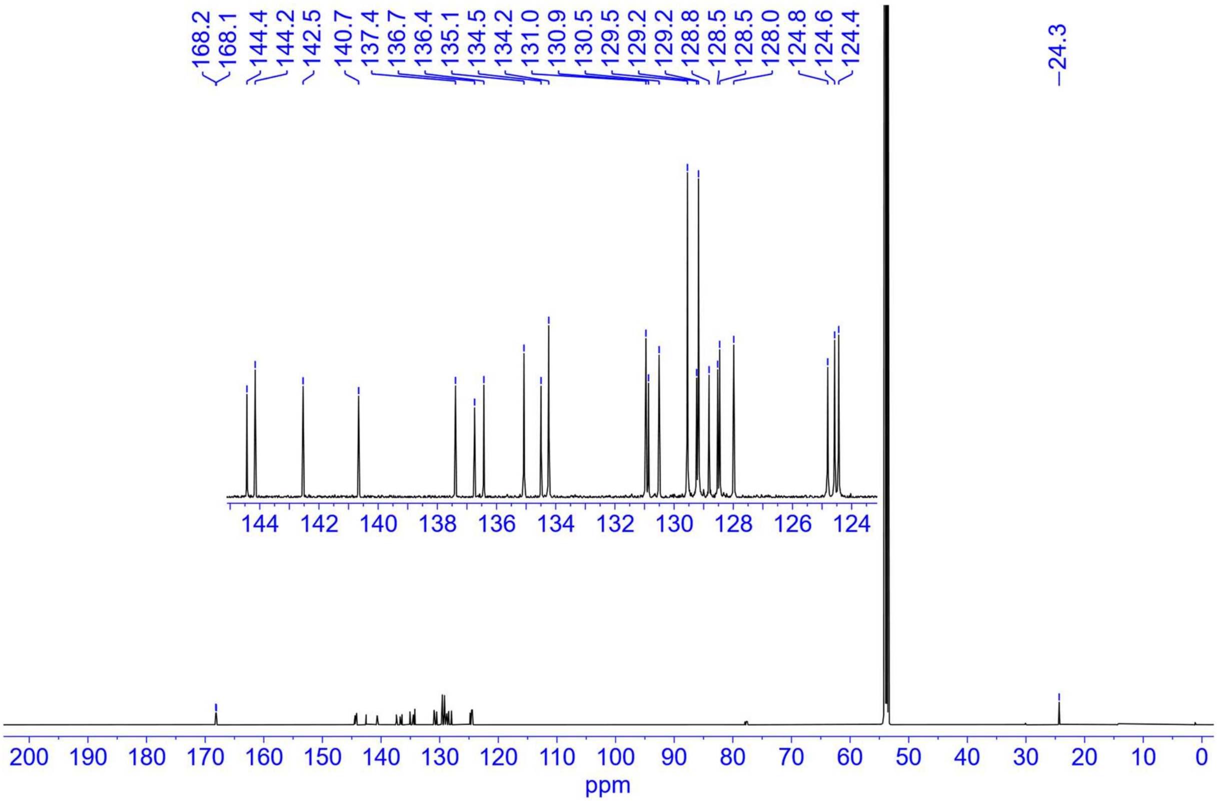

Figure S12: ^{13}C NMR (151 MHz, CD_2Cl_2) spectrum of compound **3** recorded at 298 K.

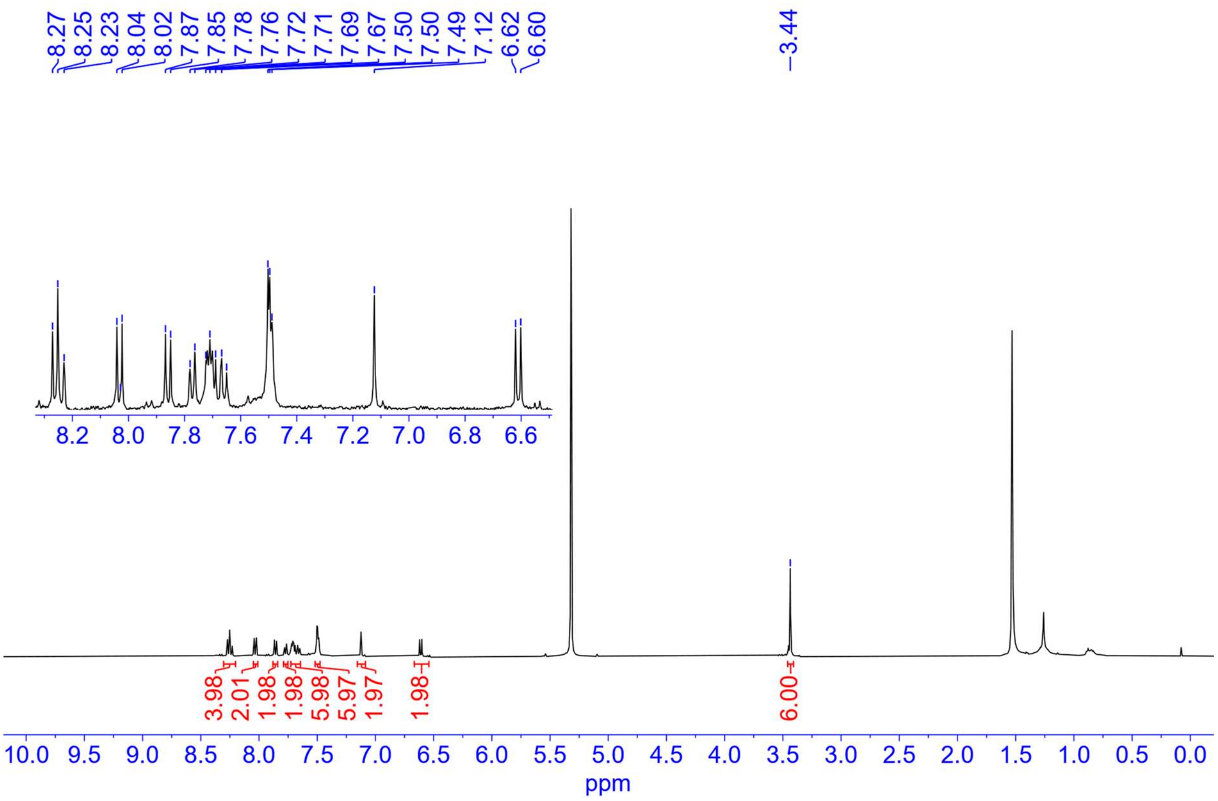

Figure S13: ^{1}H NMR (400 MHz, CD_2Cl_2) spectrum of compound **4** recorded at 298 K.

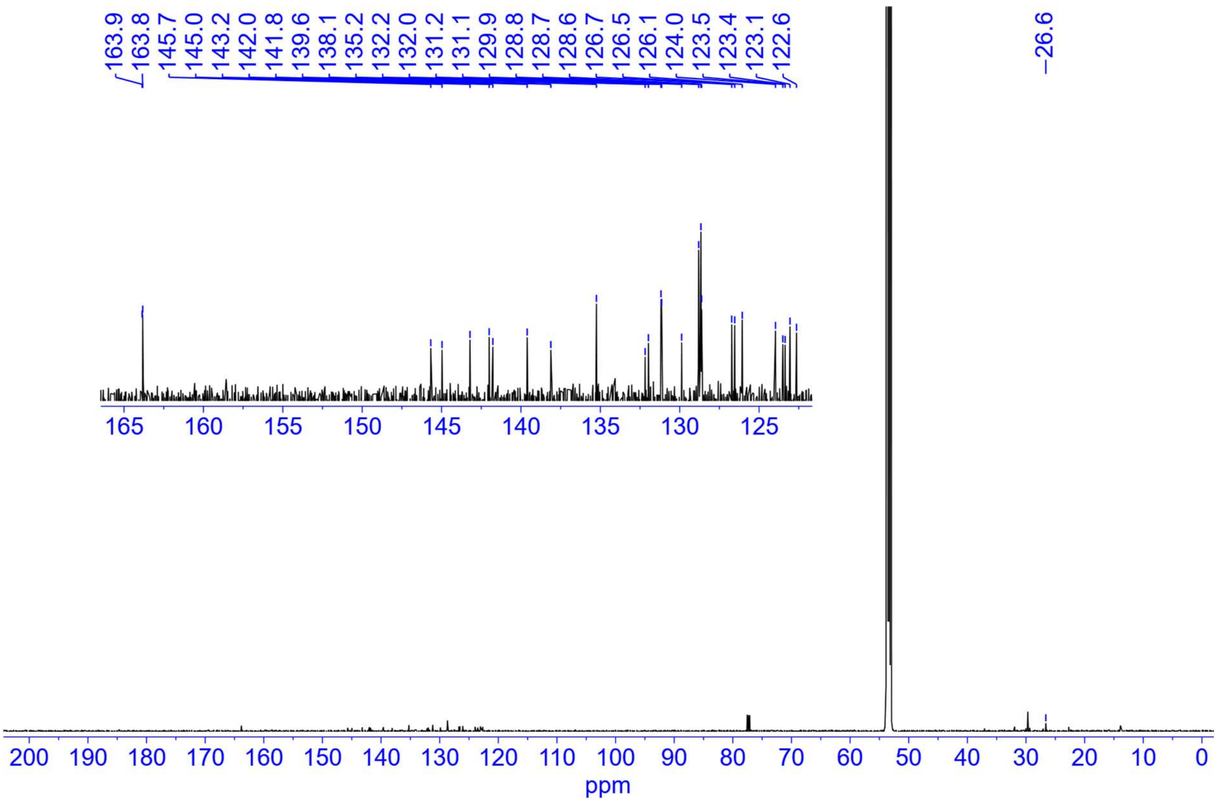

Figure S14: ^{13}C NMR (151 MHz, CD_2Cl_2) spectrum of compound **4** recorded at 298 K.

3.2 Cyclic Voltammetry

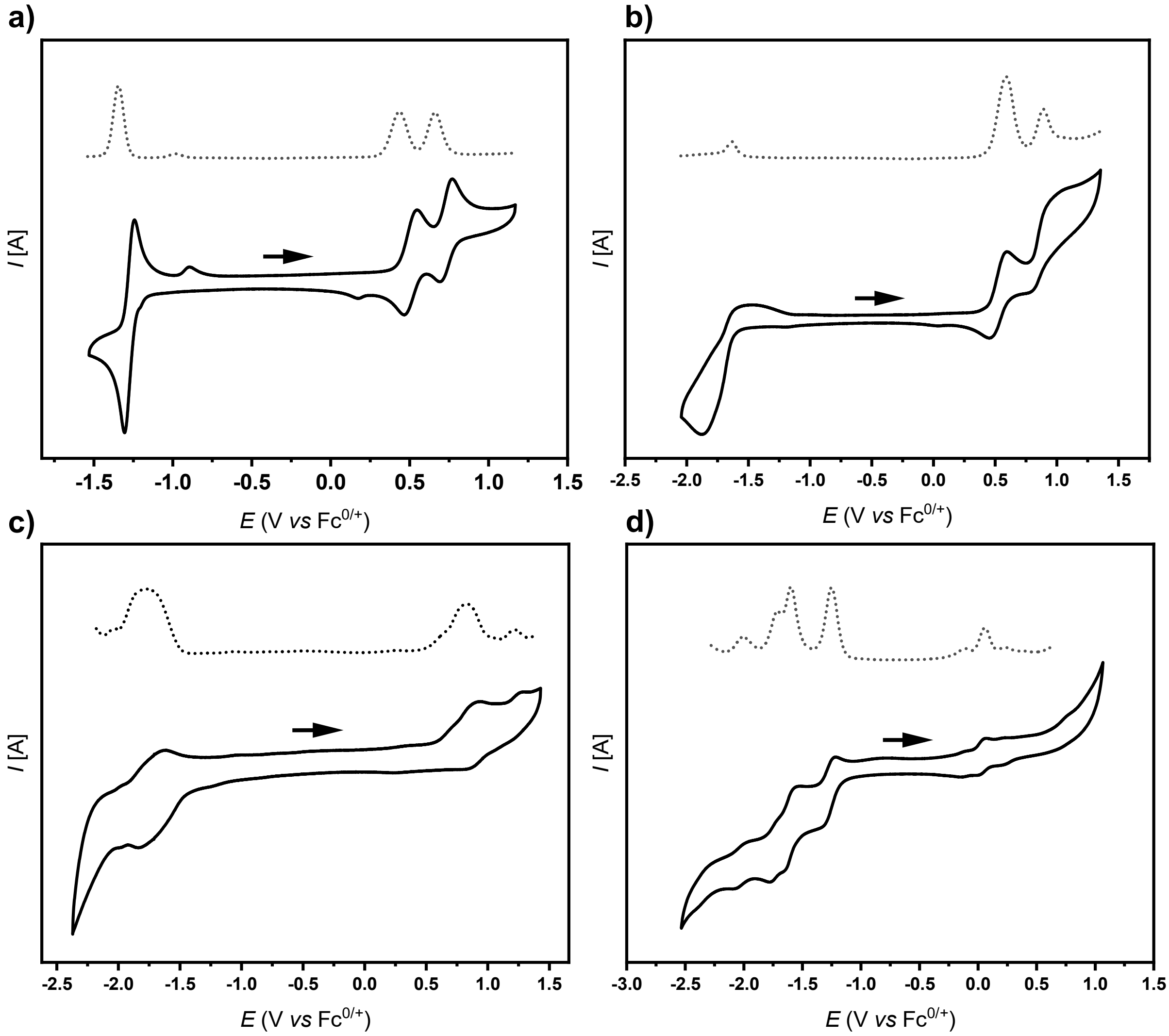

Figure S15: Differential pulse (dotted line) and cyclic (solid line) voltammograms of bisimide **1** (a), **2** (b), **3** (c), and **4** (d) measured in dry, degassed CH_2Cl_2 ($c \approx 1 \cdot 10^{-4}$ M) with 0.1 M (n-Bu)$_4$NPF$_6$ under argon atmosphere and a scan speed of 50 mVs^{-1} at 298 K (direction of scan indicated by arrows). All potentials were calculated by differential pulse voltammetry referenced against the $Fc^{0/+}$ redox couple.

3.3 UV/Vis Spectra

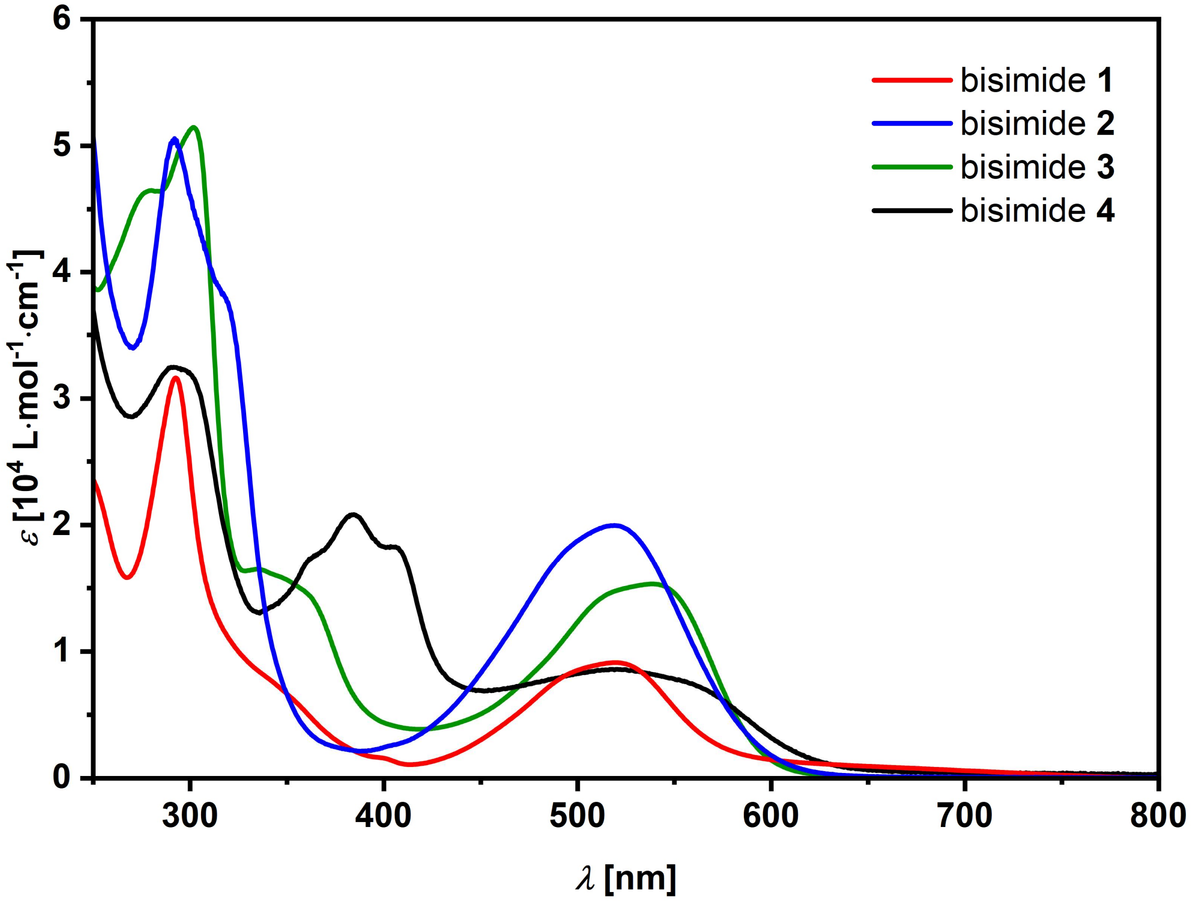

Figure S16: UV/Vis spectra of bisimides **1**–**4** ($c \approx 3 \cdot 10^{-4}$ M) measured in CH_2Cl_2 at 298 K.

4 Crystal Structure Data

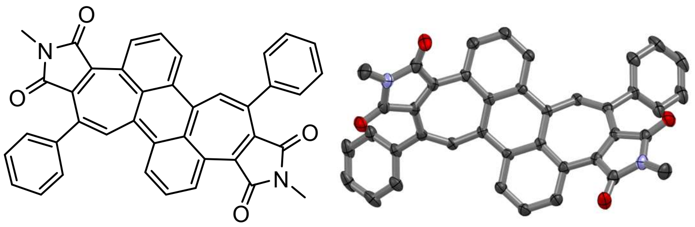

Table S1: Crystal data and structure refinement for bisimide **1**.

Empirical Formula	$C_{43.08}H_{27.08}Cl_{9.23}N_2O_4$
Formula Weight	963.85
Temperature	100(2) K
Wavelength	1.54178 Å
Crystal System	monoclinic
Space Group	*C2/c*
Z	16
Unit Cell Dimensions	*a* =18.7021(11) Å α =90°.<br>*b* =34.941(2) Å β =104.779(3)°.<br>*c* =26.6821(18) Å γ =90°.
Volume	16859.1(19) Å^3
Density (Calculated)	1.519 g·cm^{-3}
Absorption Coefficient	5.983 mm^{-1}
F(000)	7815
Crystal Size	0.340 × 0.270 × 0.120 mm^3
Theta Range for Data Collection	2.529 to 72.257°.
Index Ranges	$-22 \le h \le 15$, $-43 \le k \le 43$, $-32 \le l \le 32$
Reflections Collected	81596
Reflections (Independent)	16585 (*R*(int) = 0.1648)
Completeness to theta = 67.679°	99.8%
Absorption correction	Semi-empirical from equivalents
Max. and min. transmission	0.7536 and 0.4052
Refinement method	Full-matrix least-squares on F^2
Data / restraints / parameters	16585 / 948 / 1272
Goodness-of-fit on F^2	1.023
Final *R* Indices (I>2σ(I))	R_1 = 0.0796, wR_2 = 0.2103
R indices (all data)	R_1 = 0.0985, wR_2 = 0.2312
Largest Diff. Peak and Hole	0.753 and –0.893 e·Å^{-3}

5 Computational Data

5.1 Frontier Orbitals

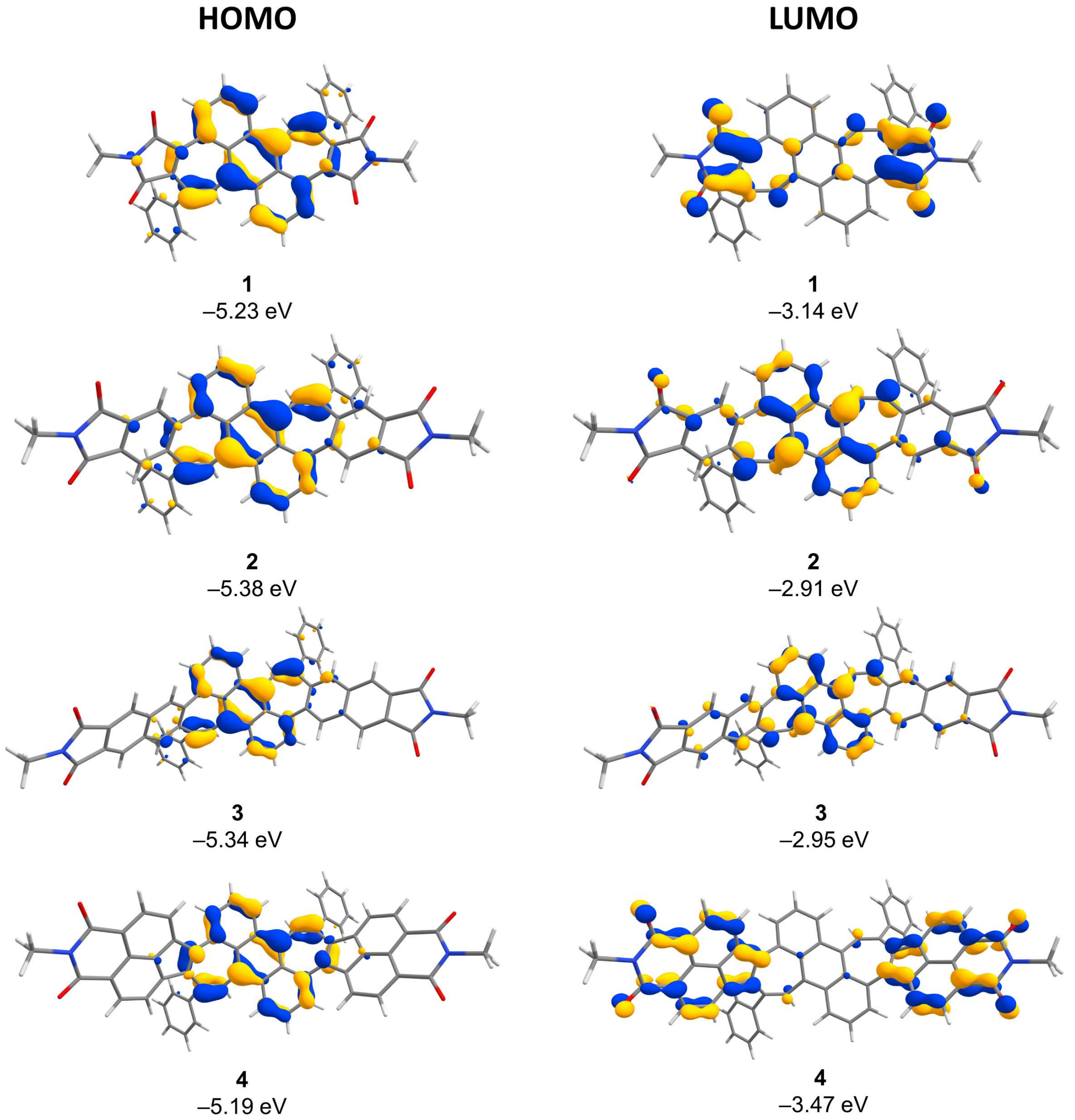

Figure S17: Frontier orbitals (HOMO left, LUMO right) of bisimide **1**–**4** calculated at the B3LYP/6-31+G(d) level of theory.

5.2 Natural Transition Orbitals

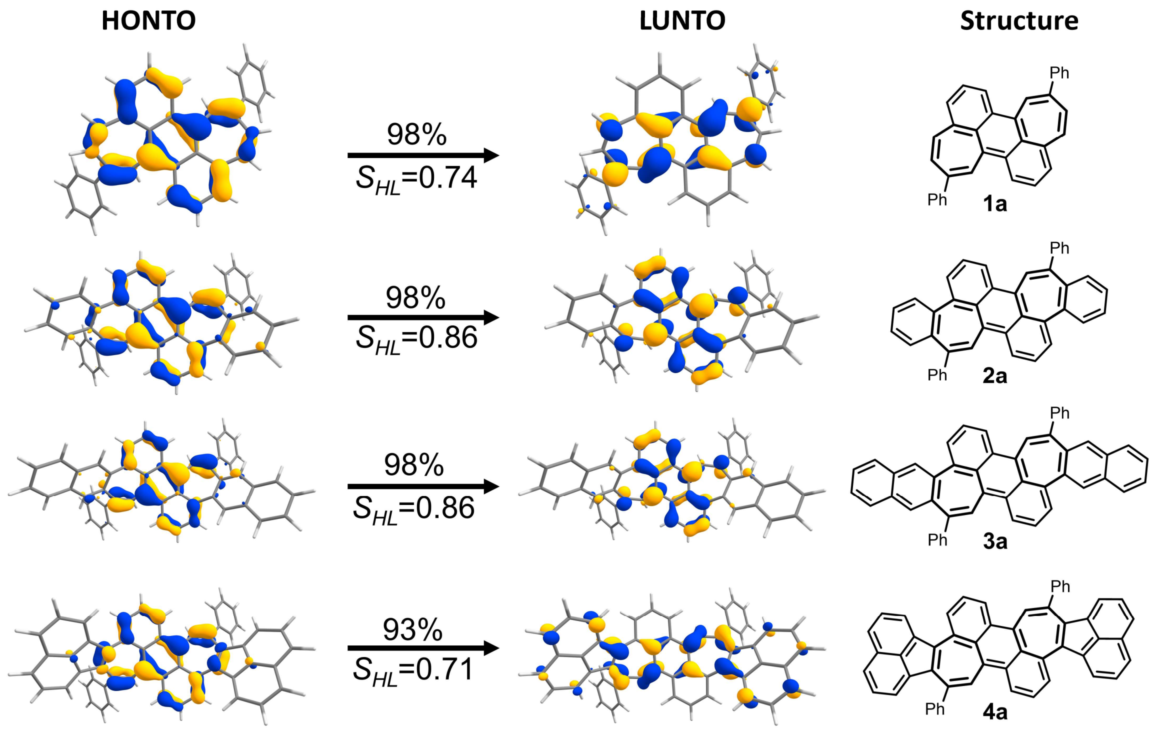

Figure S18: Structures and NTO-representations (isovalue 0.04) of the $S_0 \rightarrow S_1$ transition as HONTOs (highest occupied natural transition orbitals) and LUNTOs (lowest occupied natural transition orbitals) of PAH **1a**–**4a**. Corresponding S_{HL} values are shown below the arrows. Calculated at the CAM-B3LYP/6-31+G(d) level of theory.

5.3 Overlap Integrals S_{HL}

Table S2: Overlap integrals S_{HL} of bisimide **1**–**4** and PAH **1a**–**4a** of their respective HONTO and LUNTO calculated at the CAM-B3LYP/6-31+G(d) level of theory.

compound	S_{HL}
bisimide **1**	0.61
PAH **1a**	0.74
bisimide **2**	0.85
PAH **2a**	0.86
bisimide **3**	0.85
PAH **3a**	0.86
bisimide **4**	0.60
PAH **4a**	0.71

5.4 TD-DFT Calculations

Table S3: Excitation energies, oscillator strengths and orbital contribution of the $S_0 \rightarrow S_n$ excitations of bisimide **1**–**4** calculated at the CAM-B3LYP/6-31+G(d) level of theory.

	transition	energy [eV]	energy [nm]	oscillator strength	orbital contribution	
bisimide **1**	$S_0 \rightarrow S_1$	2.01	618	0.073	154 → 157	0.139
					155 → 156	0.677
	$S_0 \rightarrow S_2$	2.15	576	0.002	154 → 156	0.151
					155 → 157	0.674
	$S_0 \rightarrow S_3$	2.51	459	0.449	155 → 158	0.699
	$S_0 \rightarrow S_4$	3.64	341	0.013	151 → 156	–0.167
					151 → 158	0.368
					153 → 158	0.133
					155 → 159	0.308
					155 → 160	0.438
	$S_0 \rightarrow S_5$	3.67	338	0.002	146 → 157	–0.344
					147 → 156	0.298
					147 → 158	0.135
					152 → 157	0.122
					153 → 156	–0.282
					154 → 157	0.298
bisimide **2**	$S_0 \rightarrow S_1$	2.46	504	0.662	181 → 182	0.695
	$S_0 \rightarrow S_2$	2.94	422	0.024	180 → 184	0.118
					181 → 183	0.658
					181 → 187	–0.162
	$S_0 \rightarrow S_3$	3.03	409	0.007	180 → 183	0.146
					181 → 184	0.655
					181 → 189	–0.127
					181 → 190	0. 103
	$S_0 \rightarrow S_4$	3.63	342	0.004	177 → 182	–0.414
					179 → 182	0.183
					181 → 188	0.496
	$S_0 \rightarrow S_5$	3.89	318	0.006	178 → 182	0.210
					178 → 183	–0.114
					179 → 184	–0.114
					180 → 182	0.407
					181 → 185	0.459
bisimide **3**	$S_0 \rightarrow S_1$	2.43	510	0.867	207 → 208	0.692
	$S_0 \rightarrow S_2$	3.23	348	0.029	207 → 209	0.607
					207 → 213	0.278
	$S_0 \rightarrow S_3$	3.36	369	0.005	204 → 209	–0.121
					206 → 209	0.106
					207 → 210	0.594
					207 → 211	–0.122
					207 → 214	0.225
					207 → 216	0.115
	$S_0 \rightarrow S_4$	3.50	354	0.032	204 → 208	–0.124
					204 → 212	–0.104
					205 → 210	0.146
					206 → 209	–0.125

					207 → 210	0.130
					207 → 211	0.603
	$S_0 \rightarrow S_5$	3.60	344	0.003	203 → 208	–0.402
					203 → 212	0.105
					205 → 208	0.199
					207 → 212	–0.218
					207 → 215	0.435
bisimide **4**	$S_0 \rightarrow S_1$	1.79	694	0.083	218 → 221	–0.197
					219 → 220	0.644
					219 → 222	–0.126
					219 → 223	0.118
	$S_0 \rightarrow S_2$	1.87	663	0.007	218 → 220	–0.220
					219 → 221	0.649
					219 → 224	0.129
	$S_0 \rightarrow S_3$	2.35	528	0.625	219 → 220	0.117
					219 → 222	0.686
	$S_0 \rightarrow S_4$	3.04	408	0.078	214 → 220	–0.106
					215 → 220	0.353
					217 → 220	–0.109
					218 → 221	0.473
					219 → 220	0.153
					219 → 223	0.217
	$S_0 \rightarrow S_5$	3.04	408	0.000	215 → 221	0.338
					218 → 220	0.505
					219 → 221	0.202
					219 → 224	0.121

5.5 NICS Data

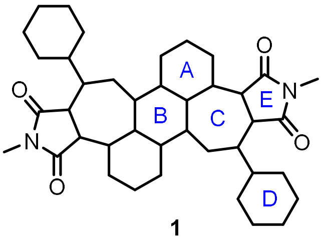

1

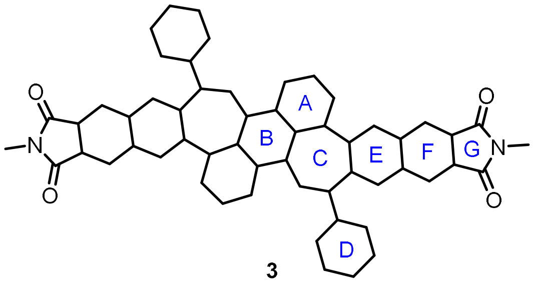

3

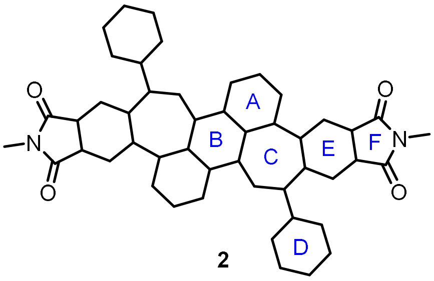

2

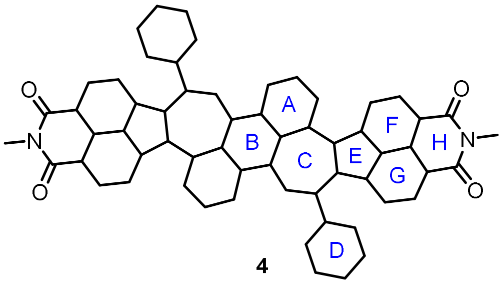

4

Table S4: $NICS_{zz}$ values of bisimides **1**–**4** calculated at the GIAO-B3LYP/6-31+G(d) level of theory. Since the compounds are non-planar, $NICS_{zz}(1)$ and $NICS_{zz}(-1)$ are averaged to $NICS_{zz}$(avg.).

		A	B	C	D	E	F	G	H
bisimide **1**	$NICS_{zz}(1)$	−22	−20	19	−27	−1			
	$NICS_{zz}(-1)$	−18	−28	20	−26	−3			
	$NICS_{zz}$(avg.)	−20	−24	19	−26	−2			
bisimide **2**	$NICS_{zz}(1)$	−23	−21	12	−26	−20	5		
	$NICS_{zz}(-1)$	−20	−29	13	−26	−20	6		
	$NICS_{zz}$(avg.)	−22	−25	13	−26	−20	6		
bisimide **3**	$NICS_{zz}(1)$	−23	−20	12	−26	−23	−21	6	
	$NICS_{zz}(-1)$	−20	−28	14	−26	−23	−21	7	
	$NICS_{zz}$(avg.)	−21	−24	13	−26	−23	−21	6	
bisimide **4**	$NICS_{zz}(1)$	−21	−27	16	−26	13	−17	−18	10
	$NICS_{zz}(-1)$	−18	−16	16	−27	15	−18	−19	10
	$NICS_{zz}$(avg.)	−20	−21	16	−27	14	−18	−19	10

5.6 NICS(1.7)-*XY*-Scan

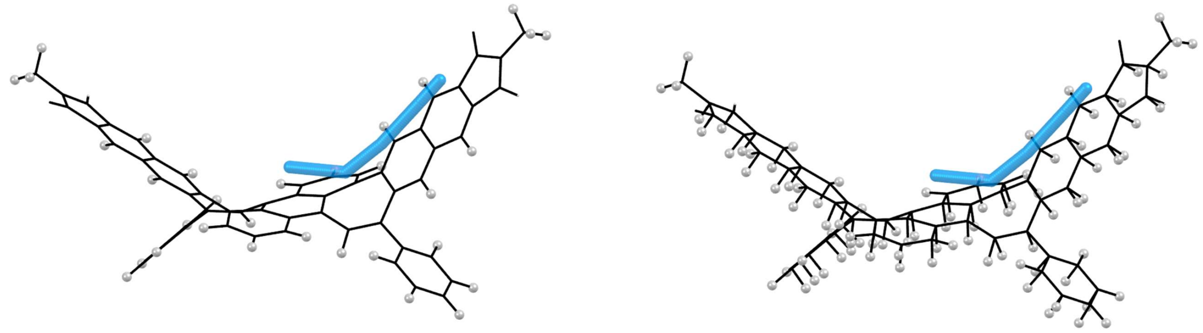

Figure S19: Representation of the NICS-*XY*-scan trajectories of bisimide **3**. Shown on the left is the unchanged molecule from which the contribution of the σ-skeleton obtained by the σ-only model (right) is subtracted, yielding uncontaminated $NICS_{\pi,zz}$ values.

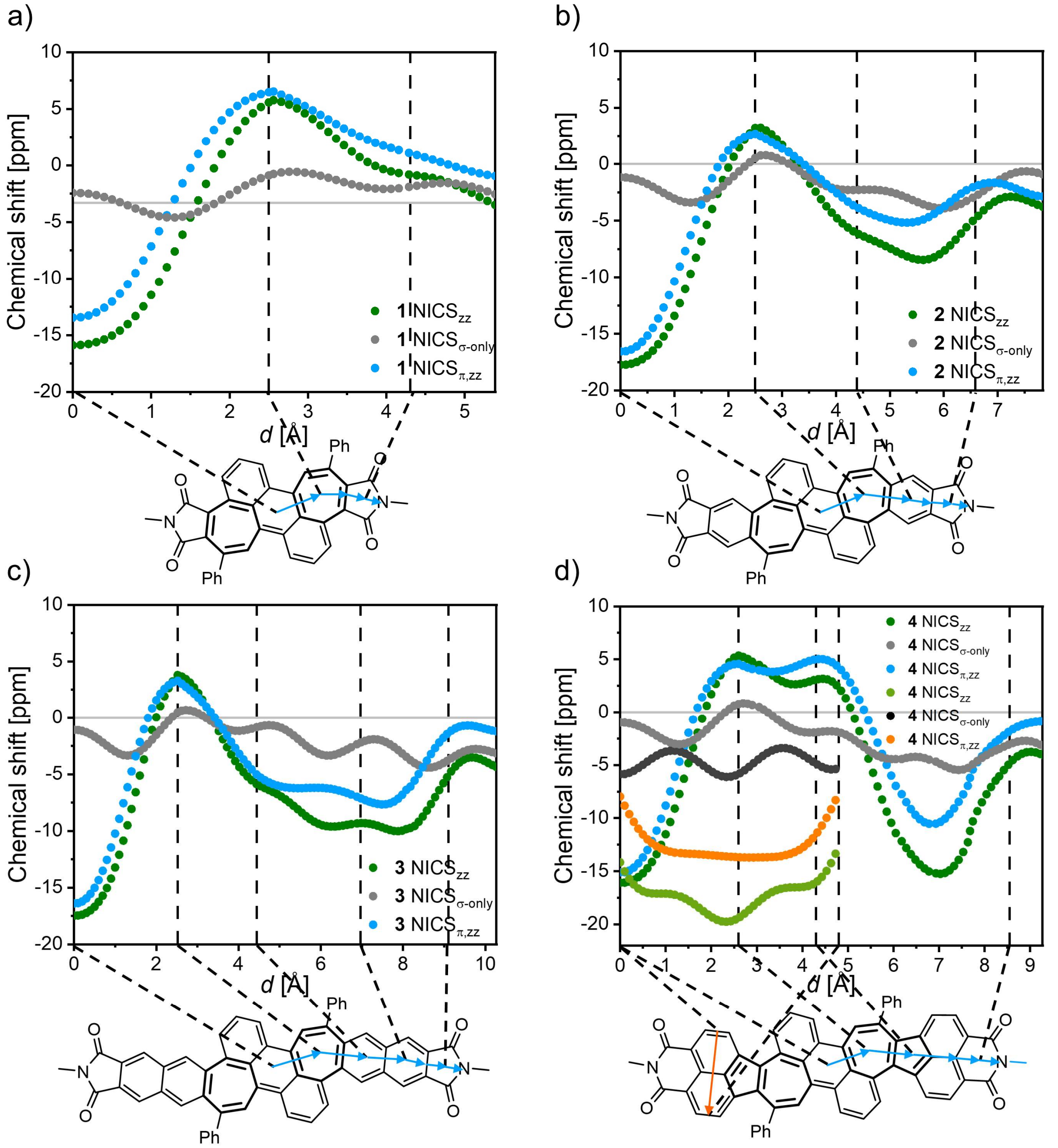

Figure S20: Subtraction of the NICS values of the σ-only model ($NICS_{σ\text{-only}}$) from those of the unmodified molecule ($NICS_{zz}$) yields $NICS_{π,zz}$ values free of σ-contamination.

5.7 ACID Plots

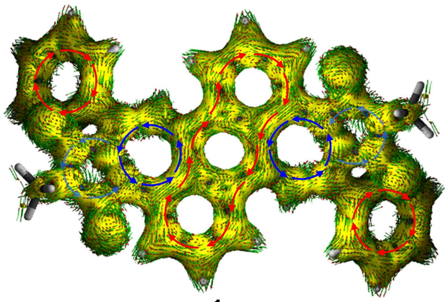

1

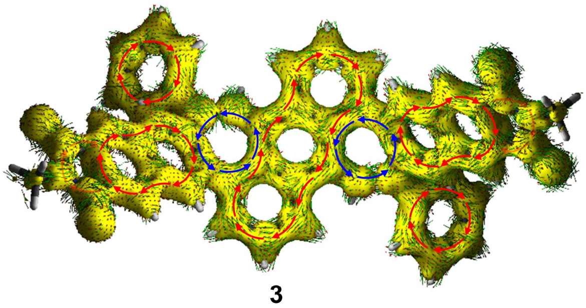

3

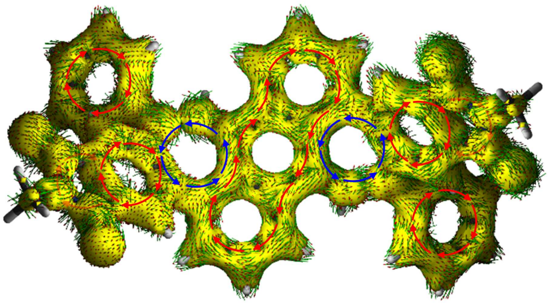

2

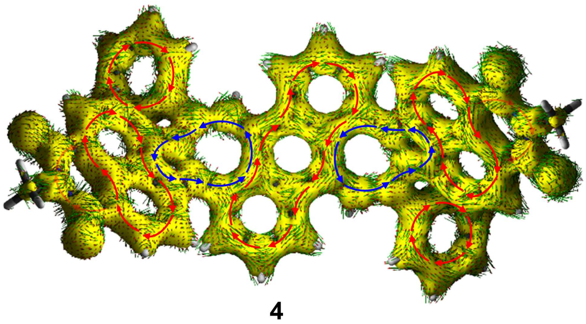

4

Figure S21: ACID plots (isovalue: 0.05) of the synthesized bisimides calculated at the B3LYP/6-31+G(d) level of theory. Diamagnetic ring currents are denoted by red, clockwise pointing arrows and paramagnetic ring currents by blue, anticlockwise pointing arrows.

5.8 Bond Lengths

Table S5: Selected bond lengths of bisimide **1**–**4** calculated at the B3LYP/6-31+G(d) level of theory.

		Bond Length [Å]			
Bond	**Ring**	imide 1	imide **2**	imide **3**	imide **4**
C1-C2	**B**	1.429	1.426	1.427	1.429
C2-C3	**A**	1.429	1.428	1.428	1.428
C3-C4	**A**	1.372	1.372	1.372	1.373
C4-C5	**A**	1.410	1.413	1.413	1.410
C5-C6	**A**	1.387	1.385	1.386	1.388
C6-C7	**A,C**	1.453	1.444	1.444	1.450
C7-C2	**A,B**	1.445	1.445	1.446	1.446
C7-C8	**B,C**	1.425	1.421	1.420	1.425
C8-C9	**C**	1.464	1.461	1.461	1.460
C9-C10	**C**	1.362	1.359	1.359	1.364
C10-C11	**C**	1.452	1.475	1.474	1.452
C11-C12	**C,E**	1.359	1.422	1.436	1.388
C12-C6	**C**	1.463	1.492	1.492	1.469
**C13-C14**	**E**	**1.518**	**1.491**	**1.489**	**1.482**
C-14-N15	**E**	1.390	1.405	1.405	1.413
N15-C16	**E**	1.396	1.405	1.405	1.413
**C16-C17**	**E**	**1.510**	**1.493**	**1.490**	**1.482**
**C13-C17**	**E/F/G/H**	**1.359**	**1.396**	**1.419**	
C17-18	**E**		1.380		
C18-C11	**E**		1.417		
C12-C19	**E**		1.416		
C19-C13	**E**		1.382		
C17-C21	**F**			1.369	
C21-C22	**F**			1.429	
C22-C23	**E**			1.412	
C23-C11	**E**			1.393	
C12-C18	**E**			1.391	
C18-C19	**E**			1.414	
C19-C20	**F**			1.427	
C20-C13	**F**			1.370	
C19-C22	**E,F**			1.436	
C17-C18	**G**				1.391
C18-C19	**G**				1.425
C19-C20	**G**				1.390
C20-C11	**E**				1.487
C12-C21	**E**				1.487
C21-C22	**F**				1.390
C22-C23	**F**				1.424
C23-C13	**F**				1.391
C21-C24	**E,F**				1.412
C24-C20	**E,G**				1.410
C24-C25	**F,G**				1.387
C25-C13	**F,H**				1.416
C25-C17	**G,H**				1.415

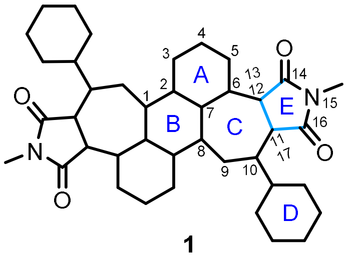

1

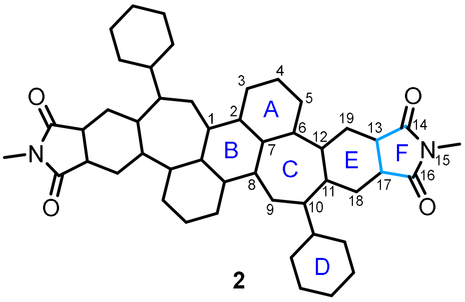

2

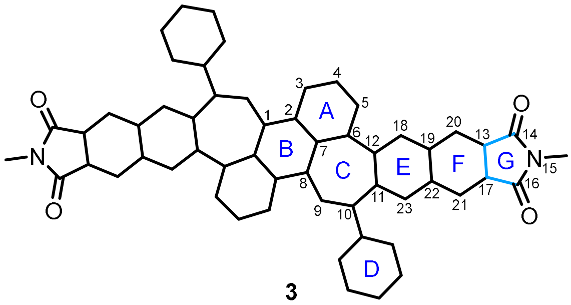

3

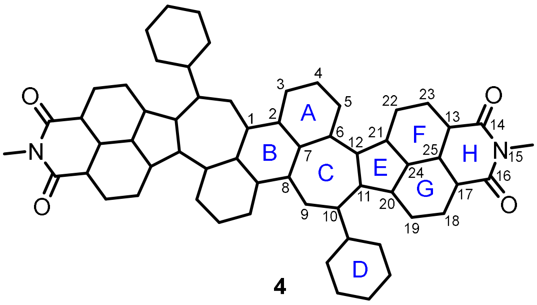

4

5.9 Optimized Structures

Table S6: Cartesian coordinates of the *cisoid* optimized geometry of bisimide **1** at the B3LYP/6-31+G(d) level of theory**.**

C	2.11292115	1.94451614	-0.31946202	C	-6.46876747	-2.13713015	-2.90870721
C	1.77380713	3.28927424	-0.32478902	C	-4.24124331	-2.37278717	-1.77012413
H	2.53583018	4.02873029	-0.53574104	H	-7.31891951	-1.46119910	-2.80330620
C	0.44008203	3.70881427	-0.14192301	C	4.97889236	-1.19822109	1.34550810
H	0.20268701	4.76850734	-0.17359601	C	5.03609836	-2.59322219	1.49972711
C	-0.56058604	2.77922520	-0.01300500	H	4.32439631	-3.22072223	0.96953807
H	-1.59215612	3.11229222	0.00917900	C	6.01999542	-3.18497023	2.29354616
C	-0.28308002	1.37793210	0.01645000	H	6.05443745	-4.26722531	2.39004417
C	1.09153208	0.94101507	-0.07097201	C	6.96538751	-2.39172917	2.94895121
C	-1.35706910	0.44307703	0.13941001	H	7.73353755	-2.85200920	3.56490226
C	-1.09152008	-0.94102007	-0.07099100	C	6.92165752	-1.00226907	2.80003520
C	0.28309302	-1.37793710	0.01643500	H	7.65245654	-0.37612103	3.30554924
C	1.35707310	-0.44308903	0.13940201	C	5.94060545	-0.41272103	2.00349214
C	-2.11291115	-1.94452014	-0.31947702	H	5.91076244	0.66950905	1.90152214
C	-1.77381213	-3.28927524	-0.32476402	C	-3.46001025	-1.56785111	-0.74746806
H	-2.53583518	-4.02873629	-0.53569604	C	-4.23172430	-0.50821904	-0.38922203
C	-0.44008303	-3.70881827	-0.14188101	C	-3.90053628	0.55048604	0.54746404
H	-0.20269501	-4.76851434	-0.17352001	C	-2.60762819	0.94568207	0.70980605
C	0.56059004	-2.77923520	-0.01298800	H	-2.45920718	1.75523612	1.42132710
H	1.59215812	-3.11230723	0.00921500	H	-6.77883449	-3.16506623	-2.70146120
C	3.46002925	1.56788011	-0.74742105	O	-3.91351028	-3.38881725	-2.35632917
C	4.23172331	0.50820604	-0.38924603	H	-6.07965545	-2.08498515	-3.93046528
C	3.90053728	-0.55052504	0.54742304	C	-5.51728042	-0.56054304	-1.17881008
C	2.60763619	-0.94570807	0.70979805	O	-6.43242045	0.23907402	-1.19161109
H	2.45920818	-1.75524413	1.42133710	C	-4.97888936	1.19817409	1.34554910
C	4.24132131	2.37290917	-1.76997013	C	-5.03607036	2.59316719	1.49984711
O	3.91364528	3.38903325	-2.35605117	H	-4.32432631	3.22068523	0.96973407
O	6.43230546	-0.23918802	-1.19183808	C	-6.01999145	3.18489123	2.29365416
C	6.46879647	2.13727315	-2.90860621	H	-6.05440443	4.26714031	2.39022217
C	5.51724941	0.56052004	-1.17887409	C	-6.96544450	2.39163617	2.94895221
H	7.31855850	1.46075511	-2.80385320	H	-7.73361556	2.85189721	3.56489125
H	6.77959348	3.16489123	-2.70083420	C	-6.92176150	1.00218507	2.79992920
H	6.07933344	2.08601615	-3.93026929	H	-7.65261857	0.37602603	3.30534724
N	5.45544437	1.72050313	-1.95345814	C	-5.94068244	0.41266003	2.00340315
N	-5.45538637	-1.72038913	-1.95357514	H	-5.91087944	-0.66956205	1.90133914

Table S7: Cartesian coordinates of the *transoid* optimized geometry of bisimide **1** at the B3LYP/6-31+G(d) level of theory**.**

C	2.11548640	1.83396338	0.77741157	C	-6.42002284	-3.54110775	1.28303270
C	1.76913205	2.95011548	1.52206784	C	-4.21899747	-3.04429324	0.17927378
H	2.53009874	3.67531980	1.77973486	H	-7.27467897	-2.94104534	1.59962637
C	0.43203558	3.19002990	1.90313346	C	5.03836797	-1.65368897	0.29626815
H	0.19544256	4.04166266	2.53465902	C	5.09854683	-2.88394295	-0.37890739
C	-0.56559790	2.38043393	1.42674934	H	4.37048974	-3.10547243	-1.15493272
H	-1.59340255	2.59204933	1.69844710	C	6.10458615	-3.80816045	-0.09243649
C	-0.28233137	1.22015775	0.63759027	H	6.14051501	-4.74964597	-0.63471761
C	1.09452114	0.85401688	0.41918507	C	7.06909302	-3.51933360	0.87647706
C	-1.36740489	0.42681787	0.13962299	H	7.85405291	-4.23757669	1.09844793
C	-1.09449770	-0.85393657	-0.41929099	C	7.02226909	-2.29660222	1.55284489
C	0.28235885	-1.22010398	-0.63765223	H	7.76763546	-2.06231810	2.30862800
C	1.36743491	-0.42677899	-0.13961367	C	6.01925013	-1.37244531	1.26249019
C	-2.11545595	-1.83382794	-0.77768583	H	5.98740281	-0.42811196	1.80076802
C	-1.76908215	-2.94989334	-1.52246926	C	-3.46165745	-1.78423089	-0.20739156
H	-2.53007268	-3.67501462	-1.78030356	C	-4.24471080	-0.72560184	0.12660451

C	-0.43197751	-3.18981662	-1.90347539	C	-3.93772349	0.68265748	-0.03932800
H	-0.19537429	-4.04137761	-2.53509290	C	-2.65028191	1.12242449	0.01078136
C	0.56564658	-2.38030643	-1.42691709	H	-2.53650595	2.19461344	-0.12778488
H	1.59345859	-2.59190985	-1.69857852	H	-6.00760490	-4.07911023	2.14249078
C	3.46166111	1.78426975	0.20705768	O	-3.87610085	-4.20797334	0.07188401
C	4.24475422	0.72557868	-0.12666320	H	-6.73113926	-4.26834547	0.52804007
C	3.93776274	-0.68263208	0.03952423	C	-5.51075302	-1.24169264	0.76416985
C	2.65030081	-1.12238094	-0.01053916	O	-6.42537515	-0.60974213	1.25539852
H	2.53649253	-2.19452673	0.12830094	C	-5.03839311	1.65369349	-0.29585935
C	4.21894016	3.04426710	-0.17993360	C	-5.09863997	2.88380845	0.37955853
O	3.87605867	4.20796630	-0.07270429	H	-4.37056320	3.10524792	1.15559146
O	6.42560772	0.60952620	-1.25508725	C	-6.10476978	3.80799898	0.09331109
C	6.42015793	3.54088258	-1.28345699	H	-6.14075436	4.74937319	0.63578152
C	5.51082060	1.24157142	-0.76427556	C	-7.06929009	3.51928238	-0.87562222
H	7.27467860	2.94066031	-1.60009844	H	-7.85432999	4.23749489	-1.09740907
H	6.73141565	4.26785594	-0.52825710	C	-7.02237725	2.29669933	-1.55225072
H	6.00797098	4.07919924	-2.14282241	H	-7.76774294	2.06250905	-2.30806392
N	5.43062431	2.63484216	-0.72429243	C	-6.01926770	1.37256750	-1.26212251
N	-5.43077084	-2.63496564	0.72352814	H	-5.98734534	0.42835706	-1.80061318

Table S8: Cartesian coordinates of the *cisoid* optimized geometry of bisimide **2** at the B3LYP/6-31+G(d) level of theory**.**

C	-2.33601824	-0.41992643	-1.63482838	H	-3.97670695	-1.85364396	3.59903346
C	-2.20006392	-0.55658841	-3.00652663	C	-5.65956756	-3.19402994	3.65318495
H	-3.06507054	-0.40123488	-3.64468290	H	-5.59084193	-3.34624219	4.72737358
C	-0.94929435	-0.83004837	-3.60540845	C	-6.66928220	-3.82116779	2.91898622
H	-0.87935345	-0.91425095	-4.68647719	H	-7.38526420	-4.47046801	3.41587359
C	0.18082726	-0.90032560	-2.82992675	C	-6.75778461	-3.59905131	1.54121777
H	1.15049542	-0.98897142	-3.30701840	H	-7.53948820	-4.08163255	0.96007691
C	0.10784460	-0.81269440	-1.40697264	C	-5.84404891	-2.75857864	0.90461665
C	-1.19169225	-0.67037626	-0.79040171	H	-5.91925076	-2.59638319	-0.16762070
C	1.28816371	-0.88631342	-0.61059312	C	3.58820207	0.21173548	1.12486128
C	1.19167019	-0.67032386	0.79040738	C	4.30445156	-0.21484193	-0.02698931
C	-0.10786565	-0.81261669	1.40698626	C	3.81043701	-1.27752145	-0.92318978
C	-1.28818431	-0.88628831	0.61061085	C	2.49337610	-1.45981034	-1.20359666
C	2.33599443	-0.41981879	1.63482005	H	2.28165854	-2.21780111	-1.95479458
C	2.20004122	-0.55640579	3.00652588	C	4.08057297	1.30552568	1.87726287
H	3.06504669	-0.40101046	3.64467352	H	3.52063318	1.67697766	2.72902160
C	0.94927368	-0.82984153	3.60542294	C	5.26507568	1.91344497	1.50820677
H	0.87933344	-0.91398469	4.68649636	C	5.98206516	1.47135835	0.39546772
C	-0.18084752	-0.90016953	2.82994523	C	5.52218949	0.42182765	-0.37344692
H	-1.15051501	-0.98879569	3.30704192	H	6.07842019	0.09578720	-1.24570894
C	-3.58823063	0.21164670	-1.12490467	C	5.99592490	3.06592132	2.10979145
C	-4.30447786	-0.21487281	0.02696884	O	5.71125246	3.73333551	3.08753784
C	-3.81045499	-1.27749794	0.92322911	H	8.92139723	3.90479684	2.21448262
C	-2.49339259	-1.45976065	1.20364642	H	7.65226609	5.13849351	2.01298429
H	-2.28166947	-2.21770706	1.95488751	H	8.58320640	4.56001573	0.59274083
C	-4.08060769	1.30539350	-1.87736502	C	7.19669110	2.32770924	0.25133670
H	-3.52066882	1.67680515	-2.72914188	C	4.81364543	-2.13081666	-1.62840125
C	-5.26512051	1.91331749	-1.50834870	C	4.74229385	-2.35691936	-3.01387792
C	-5.98210834	1.47128658	-0.39558636	H	3.97669365	-1.85382016	-3.59896099
C	-5.52221992	0.42180810	0.37339171	C	5.65956507	-3.19419568	-3.65303525
H	-6.07844702	0.09581320	1.24567310	H	5.59084077	-3.34647034	-4.72721512
C	-5.99597099	3.06575542	-2.11000526	C	6.66928474	-3.82128317	-2.91880037
O	-5.71115874	3.73325048	-3.08765433	H	7.38527193	-4.47060634	-3.41565031
C	8.13564151	4.27726578	1.54754403	C	6.75778524	-3.59908669	-1.54104470
O	8.08166633	2.27563300	-0.58251645	H	7.53949260	-4.08162836	-0.95987596
N	7.13121542	3.25527863	1.30427329	C	5.84404277	-2.75858467	-0.90449203
N	-7.13116446	3.25527647	-1.30437400	H	5.91924314	-2.59632700	0.16773601
C	-7.19674089	2.32763842	-0.25151597	C	-8.13546133	4.27743514	-1.54745910

O	-8.08162082	2.27575766	0.58245208	H	-8.92248929	3.90442368	-2.21259219
C	-4.81365659	-2.13076064	1.62848967	H	-8.58139571	4.56186713	-0.59238636
C	-4.74230309	-2.35678305	3.01397936	H	-7.65229726	5.13768088	-2.01491683

Table S9: Cartesian coordinates of the *cisoid* optimized geometry of bisimide **3** at the B3LYP/6-31+G(d) level of theory.

C	-2.45115945	-1.13240641	-1.45538746	H	-7.39302988	-4.86511767	1.62018084
C	-2.41718973	-1.27037702	-2.83364583	C	-5.73519201	-3.51222786	1.39672006
H	-3.32926518	-1.12087829	-3.40383561	H	-5.88958382	-3.38951624	0.32777545
C	-1.21478929	-1.54340910	-3.52421224	C	3.66676442	-0.50899761	0.85654736
H	-1.22533547	-1.62866590	-4.60752168	C	4.30697480	-0.94877850	-0.35122491
C	-0.03059793	-1.61742495	-2.83462051	C	3.73409754	-1.99460048	-1.21843439
H	0.90023842	-1.70964901	-3.38270000	C	2.39809185	-2.17177164	-1.39425764
C	0.00228648	-1.53253444	-1.40991798	H	2.12842392	-2.92037670	-2.13589320
C	-1.24800545	-1.38830971	-0.69861173	C	4.22296378	0.56388709	1.54620957
C	1.24029871	-1.60676678	-0.70507216	H	3.71809787	0.93761293	2.43224643
C	1.24826027	-1.38872523	0.69849074	C	5.41259001	1.21279922	1.14228939
C	-0.00204813	-1.53311576	1.40973598	C	6.07713302	0.73751699	-0.03875962
C	-1.24006863	-1.60693243	0.70485987	C	5.49254604	-0.33450894	-0.74694450
C	2.45143918	-1.13324268	1.45537245	H	5.98570632	-0.67774798	-1.65116666
C	2.41744917	-1.27177129	2.83357596	C	5.95715785	2.31040187	1.87410903
H	3.32953748	-1.12260053	3.40383091	H	5.45977706	2.67879804	2.76765667
C	1.21502058	-1.54496836	3.52402694	C	7.11743977	2.88043974	1.42076430
H	1.22555637	-1.63066816	4.60730168	C	8.99436522	3.24133060	0.04835779
C	0.03082349	-1.61859016	2.83440297	C	7.77643436	2.40860270	0.25611241
H	-0.90002309	-1.71095286	3.38244189	C	7.90812350	4.01797601	1.96748899
C	-3.66641812	-0.50828031	-0.85630653	C	7.28601908	1.36041431	-0.47605630
C	-4.30668045	-0.94849056	0.35128096	H	7.79675852	1.00640128	-1.36777618
C	-3.73391036	-1.99472848	1.21806266	C	4.66892645	-2.83946636	-2.02260875
C	-2.39792132	-2.17210821	1.39380966	C	4.49455928	-3.01286043	-3.40635428
H	-2.12832662	-2.92105339	2.13512837	H	3.69823448	-2.47582517	-3.91538116
C	-4.22246559	0.56497813	-1.54550592	C	5.34665420	-3.84229614	-4.13992570
H	-3.71756870	0.93898982	-2.43140423	H	5.19758905	-3.95421236	-5.21097727
C	-5.41189086	1.21401204	-1.14119078	C	6.39259611	-4.51466057	-3.50321031
C	-6.07650213	0.73831206	0.03965282	H	7.05691941	-5.15922011	-4.07280465
C	-5.49216646	-0.33422814	0.74726775	C	6.58317490	-4.34557483	-2.12788155
H	-5.98538892	-0.67781050	1.65132555	H	7.39279598	-4.86536469	-1.62172400
C	-5.95633471	2.31196173	-1.87257594	C	5.73515889	-3.51232008	-1.39770591
H	-5.45843284	2.68123541	-2.76547114	H	5.88952032	-3.39012744	-0.32869701
C	-7.11723804	2.88112471	-1.41972498	C	-8.99476586	3.24092284	-0.04786588
C	-10.03636189	5.17928734	-1.27650102	O	-9.83667490	3.17005049	0.82952794
C	-7.77633355	2.40884559	-0.25530859	O	7.69488216	4.70261467	2.95226319
H	-9.59732194	6.04864174	-1.76978887	C	-7.90828972	4.01836236	-1.96652977
C	-7.28537110	1.36110887	0.47714194	O	-7.69607816	4.70202700	-2.95220232
H	-7.79572927	1.00730710	1.36916451	N	-9.00107989	4.17518593	-1.09717322
C	-4.66883024	-2.83982499	2.02188720	H	-10.43065080	5.45280901	-0.29588384
C	-4.49443278	-3.01388505	3.40554670	H	-10.85408389	4.79250561	-1.89515789
H	-3.69800950	-2.47720272	3.91479221	O	9.83495714	3.17222855	-0.83043986
C	-5.34662288	-3.84353118	4.13876904	H	10.13575059	5.40238085	2.33681511
H	-5.19753119	-3.95596422	5.20976289	N	9.00632605	4.16853978	1.10386452
C	-6.39269374	-4.51544289	3.50178646	C	10.03063493	5.18576133	1.27195916
H	-7.05709340	-5.16016254	4.07111023	H	9.75752930	6.10713282	0.74522352
C	-6.58330487	-4.34569162	2.12654471	H	10.96913512	4.80667833	0.86280644

Table S10: Cartesian coordinates of the *cisoid* optimized geometry of bisimide **4** at the B3LYP/6-31+G(d) level of theory.

C	-2.44980734	-0.98939171	-1.48490736	C	6.53786532	-0.05068887	-1.47748267
C	-2.38754194	-1.05105550	-2.86996916	C	5.63762041	0.72199418	0.60073451

C	-1.16853249	-1.25957030	-3.54796637	C	4.65416457	-2.68458409	-2.08971619
C	0.00236622	-1.35667330	-2.83782418	C	4.47174729	-2.81709716	-3.47727229
C	0.01022693	-1.34593728	-1.40992041	C	5.32768928	-3.61502376	-4.24006901
C	-1.25017795	-1.23925933	-0.70993140	C	6.38362391	-4.29689381	-3.62900350
C	1.24537861	-1.45502591	-0.69875076	C	5.50642948	2.36485154	2.78818261
C	1.25017618	-1.23927162	0.70991465	C	7.59673455	0.88669651	-1.30561892
C	-0.01022863	-1.34596236	1.40990174	O	-9.69004840	2.83455238	0.79535641
C	-1.24538030	-1.45503878	0.69873014	C	7.66268855	1.73285650	-0.20370981
C	2.44980551	-0.98941755	1.48489493	H	-3.29470412	-0.90416243	-3.44675702
C	2.38754011	-1.05110598	2.86995559	H	-1.15373089	-1.28033764	-4.63452333
C	1.16853066	-1.25963290	3.54794916	H	0.94526612	-1.39734661	-3.37131462
C	-0.00236805	-1.35672382	2.83780531	H	3.29470225	-0.90422301	3.44674609
C	-3.68989629	-0.49790568	-0.86941120	H	1.15372910	-1.28041943	4.63450575
C	-4.28197615	-0.90428394	0.31887445	H	-0.94526803	-1.39740700	3.37129491
C	-3.72698075	-1.86673318	1.25401767	H	-2.09077999	-2.74808202	2.17485010
C	-2.38220888	-2.02838928	1.41271538	H	-6.53248041	-0.67445377	2.36502699
C	-6.64582727	1.65820550	-0.77786485	H	-3.64369056	1.37573989	-3.27374221
C	-6.58191768	2.49728807	-1.91613794	H	-5.45579812	3.02225597	-3.65099501
C	-5.54482516	-0.14200472	0.50983052	H	-8.37670323	0.95471456	2.05805279
C	-6.53786865	-0.05071609	1.47748406	H	-3.66756286	-2.27205985	3.96490751
C	-4.47354015	1.40789958	-2.57478114	H	-5.17380538	-3.69658292	5.31311230
C	-5.50642938	2.36490060	-2.78813823	H	-7.05081693	-4.91709269	4.22182720
C	-7.65999922	3.49334137	-2.11958069	H	-7.39437563	-4.70096671	1.76401535
C	-7.59673682	0.88667270	1.30563582	H	-5.88059595	-3.28308034	0.41706513
C	-7.66269384	1.73285481	0.20374245	H	9.31808395	5.39635157	1.85410198
C	-4.65416644	-2.68462172	2.08967355	O	7.70688004	4.23586347	3.09276836
C	-4.47174872	-2.81716030	3.47722717	H	2.09077850	-2.74804265	-2.17489371
C	-5.32769103	-3.61510027	4.24000960	H	3.64369238	1.37568260	3.27377268
C	-6.38362618	-4.29695862	3.62893198	H	6.53247522	-0.67441106	-2.36503646
C	-6.57834685	-4.17178463	2.24964369	H	3.66756194	-2.27198732	-3.96494296
C	-5.72566275	-3.37034477	1.48947166	H	5.17380405	-3.69648661	-5.31317326
C	3.68989442	-0.49792057	0.86940729	H	7.05081455	-4.91701735	-4.22190984
C	4.28197453	-0.90427757	-0.31888553	H	7.39437246	-4.70093662	-1.76409392
C	3.72697915	-1.86671023	-1.25404566	H	5.88059304	-3.28307416	-0.41711825
C	2.38220723	-2.02836353	-1.41274608	H	-10.09709012	4.84567111	-0.32880533
C	5.54482341	-0.14199436	-0.50982902	H	-10.54950248	4.11620041	-1.88627849
C	4.54022139	0.56090644	1.47424621	H	-9.31716487	5.39772981	-1.85167924
C	4.47354068	1.40785403	2.57481064	H	10.09515661	4.84772822	0.32853055
C	6.57834415	-4.17174518	-2.24971284	O	9.69007566	2.83460352	-0.79521337
C	5.72566010	-3.37031894	-1.48952640	H	5.45579712	3.02219242	3.65104999
C	-4.54022289	0.56093276	-1.47423163	C	6.58191969	2.49725796	1.91618569
C	-8.75042599	2.72258857	0.01773668	C	9.72526346	4.54710294	1.30757448
C	-5.63762237	0.72200394	-0.60071705	H	8.37670181	0.95475139	-2.05803425
N	-8.65563902	3.55612204	-1.11916225	C	8.75040678	2.72258799	-0.01765503
C	-9.72515244	4.54720348	-1.30771888	N	8.65563166	3.55610801	1.11923548
O	-7.70697661	4.23590644	-3.09265454	C	7.65998530	3.49332831	2.11966947
C	6.64582510	1.65819290	0.77789850	H	10.55096475	4.11513690	1.88348289

Table S11: Cartesian coordinates of the *cisoid* optimized geometry of PAH **1a** at the B3LYP/6-31+G(d) level of theory**.**

C	-1.51724238	-2.45662583	-0.93401380	C	-7.49128465	0.85623632	1.91931760
C	-0.80019047	-3.63695424	-1.09529233	H	-8.39421154	1.19542429	2.42039780
H	-1.34775594	-4.55731389	-1.28229001	C	-7.13669843	-0.49536467	1.94239262
C	0.60563204	-3.66668436	-1.07221085	H	-7.75864881	-1.21347490	2.47122157
H	1.13159916	-4.60325586	-1.23797427	C	-5.97986468	-0.93063676	1.29348808
C	1.30571014	-2.49897114	-0.89898247	H	-5.70664735	-1.98181649	1.33664069
H	2.38691811	-2.51657501	-0.97335606	C	3.90335231	0.50440166	-0.06052470
C	0.64352237	-1.24597753	-0.71881944	C	2.77315914	-0.25786424	-0.00945368
C	-0.80259905	-1.20749111	-0.72809895	H	2.86756674	-1.17646046	0.56325528
C	1.42904391	-0.05906999	-0.53741410	C	5.14282501	0.02505454	0.61418237

C	0.80259063	1.20753485	-0.72802354	C	5.51987905	-1.33084928	0.59336022
C	-0.64353740	1.24603427	-0.71874617	H	4.91508205	-2.04634107	0.04288747
C	-1.42906610	0.05910677	-0.53741806	C	6.67713302	-1.76705763	1.23952191
C	1.51727042	2.45664938	-0.93391886	H	6.94985828	-2.81886449	1.20092005
C	0.80026222	3.63700632	-1.09512997	C	7.49125645	-0.85622907	1.91937316
H	1.34784945	4.55735343	-1.28212589	H	8.39418021	-1.19539805	2.42047170
C	-0.60556220	3.66676678	-1.07204395	C	7.13675654	0.49539776	1.94225239
H	-1.13150494	4.60335718	-1.23777759	H	7.75877248	1.21355147	2.47094543
C	-1.30567479	2.49906435	-0.89888960	C	5.97992527	0.93064326	1.29332556
H	-2.38687430	2.51672396	-0.97332293	H	5.70677694	1.98184705	1.33632651
C	-3.90336475	-0.50446172	-0.06051133	C	3.98416421	1.77747573	-0.75343420
C	-2.77319129	0.25784355	-0.00946153	H	4.98770008	2.13346089	-0.97690313
H	-2.86764418	1.17641396	0.56327164	C	2.96206358	2.58159928	-1.12078778
C	-5.14284826	-0.02509848	0.61417622	H	3.25020936	3.51410083	-1.60447664
C	-5.51998706	1.33077865	0.59316057	C	-2.96204236	-2.58166525	-1.12072598
H	-4.91525513	2.04621961	0.04255067	H	-3.25018626	-3.51422905	-1.60429418
C	-6.67724600	1.76701293	1.23929653	C	-3.98415572	-1.77758088	-0.75332249
H	-6.95004195	2.81879578	1.20054220	H	-4.98768682	-2.13366470	-0.97665116

Table S12: Cartesian coordinates of the *cisoid* optimized geometry of PAH **2a** at the B3LYP/6-31+G(d) level of theory**.**

C	2.02112859	2.01333561	-0.56563995	C	7.07327241	-1.75994192	2.81990144
C	1.64881716	3.34050571	-0.42657533	H	7.86143160	-2.13139156	3.46979038
H	2.39234906	4.11766290	-0.57735748	C	6.93035222	-0.38625051	2.59866198
C	0.31241049	3.71423291	-0.15703402	H	7.60367534	0.31683430	3.08295610
H	0.05604212	4.76720853	-0.07303309	C	5.92274360	0.09025738	1.75939512
C	-0.66705071	2.75552307	-0.08618813	H	5.81813383	1.15967876	1.59603538
H	-1.70482276	3.05722795	0.00169205	C	-3.34583675	-1.72641001	-1.19003659
C	-0.34873473	1.36627927	-0.17145475	C	-4.24475993	-0.71436162	-0.77276783
C	1.03888768	0.98420957	-0.31290651	C	-3.91552772	0.26391909	0.28220561
C	-1.37520033	0.37927536	-0.09674862	C	-2.66631106	0.76210710	0.47224426
C	-1.03888772	-0.98420952	-0.31290661	H	-2.58490385	1.53608305	1.23266559
C	0.34873469	-1.36627928	-0.17145501	C	-3.72468493	-2.54666966	-2.27360547
C	1.37520032	-0.37927536	-0.09674881	H	-3.02135712	-3.29254355	-2.63116127
C	-2.02112867	-2.01333547	-0.56564025	C	-5.49297785	-0.60398754	-1.42107695
C	-1.64881731	-3.34050560	-0.42657586	H	-6.18362142	0.16620265	-1.09241606
H	-2.39234925	-4.11766274	-0.57735819	C	-5.02505343	0.79322702	1.13151425
C	-0.31241066	-3.71423289	-0.15703458	C	-5.18703340	2.17143872	1.35647316
H	-0.05604232	-4.76720854	-0.07303381	H	-4.52716356	2.87461186	0.85478470
C	0.66705060	-2.75552311	-0.08618861	C	-6.19883341	2.65062512	2.19217739
H	1.70482264	-3.05722809	0.00169147	H	-6.30849323	3.72164686	2.34426514
C	3.34583679	1.72641044	-1.19003620	C	-7.07327262	1.75994064	2.81990199
C	4.24476009	0.71436211	-0.77276748	H	-7.86143186	2.13138993	3.46979104
C	3.91552777	-0.26391904	0.28220552	C	-6.93035259	0.38624937	2.59866152
C	2.66631110	-0.76210720	0.47224388	H	-7.60367592	-0.31683568	3.08295498
H	2.58490398	-1.53608349	1.23266488	C	-5.92274392	-0.09025806	1.75939452
C	3.72468502	2.54667037	-2.27360485	H	-5.81813430	-1.15967934	1.59603401
H	3.02135716	3.29254425	-2.63116057	C	5.85185967	1.43588867	-2.47599486
C	5.49297817	0.60398849	-1.42107632	H	6.81626060	1.31222949	-2.96181372
H	6.18362190	-0.16620155	-1.09241539	C	4.95445858	2.41354145	-2.91222021
C	5.02505339	-0.79322740	1.13151402	H	5.20397896	3.06169838	-3.74832909
C	5.18703355	-2.17143925	1.35647191	C	-4.95445833	-2.41354037	-2.91222104
H	4.52716394	-2.87461210	0.85478278	H	-5.20397874	-3.06169714	-3.74833003
C	6.19883352	-2.65062609	2.19217599	C	-5.85185930	-1.43588743	-2.47599572
H	6.30849344	-3.72164793	2.34426297	H	-6.81626012	-1.31222798	-2.96181474

Table S13: Cartesian coordinates of the *cisoid* optimized geometry of PAH **3a** at the B3LYP/6-31+G(d) level of theory**.**

C	2.26277460	-0.06993789	1.73594957	H	6.13483673	0.40501292	-0.93715421
C	2.06461409	-0.21340245	3.09989338	H	7.48695022	-4.09818112	-3.20622567
H	2.90318387	-0.06656494	3.77409625	C	6.79510759	-3.26866395	-1.33411338
C	0.78798971	-0.48487412	3.64241344	H	7.54375787	-3.78146298	-0.73512585
H	0.66979817	-0.57178516	4.71943624	C	5.86813065	-2.43229785	-0.71137479
C	-0.30650255	-0.55646028	2.81763188	H	5.90126895	-2.29883753	0.36673975
H	-1.29622040	-0.64818215	3.25062242	C	-3.54033339	0.55757638	-1.28769069
C	-0.16959998	-0.46990190	1.39908100	C	-4.31988820	0.12291419	-0.15986901
C	1.15693487	-0.32539015	0.84185032	C	-3.85306438	-0.92239247	0.77020167
C	-1.31546937	-0.54326840	0.55263443	C	-2.54799472	-1.10618359	1.10113573
C	-1.15693428	-0.32538411	-0.84185195	H	-2.36799533	-1.85929342	1.86518300
C	0.16960056	-0.46989246	-1.39908360	C	-4.01053299	1.62620699	-2.04119979
C	1.31546990	-0.54326509	-0.55263748	H	-3.40603225	1.99493386	-2.86534437
C	-2.26277394	-0.06992560	-1.73594951	C	-5.23885454	2.28016997	-1.77712452
C	-2.06461342	-0.21338060	-3.09989430	C	-6.03325115	1.81453221	-0.68385296
H	-2.90318315	-0.06653807	-3.77409615	C	-5.53904294	0.74158442	0.09372008
C	-0.78798905	-0.48484871	-3.64241619	H	-6.13483720	0.40500618	0.93715617
H	-0.66979746	-0.57175222	-4.71943960	C	-5.70797098	3.37399113	-2.55585237
C	0.30650314	-0.55644092	-2.81763508	H	-5.09998252	3.72854149	-3.38545272
H	1.29622096	-0.64815992	-3.25062624	C	-7.27427917	2.45464970	-0.40900149
C	3.54033392	0.55756720	1.28769485	H	-7.87701576	2.09756326	0.42319508
C	4.31988826	0.12291306	0.15986983	C	-4.87634364	-1.76854474	1.45715549
C	3.85306468	-0.92238790	-0.77020745	C	-4.86062581	-1.95433181	2.85032271
C	2.54799509	-1.10617709	-1.10114251	H	-4.12470433	-1.42492680	3.45010841
H	2.36799551	-1.85928236	-1.86519422	C	-5.79198607	-2.78683313	3.47643586
C	4.01053362	1.62619282	2.04121102	H	-5.76408996	-2.90838144	4.55657342
H	3.40603361	1.99491338	2.86535894	C	-6.76146461	-3.45052017	2.72067793
C	5.23885433	2.28015858	1.77713906	H	-7.48694926	-4.09820063	3.20620133
C	6.03325036	1.81452881	0.68386365	C	-6.79510663	-3.26867265	1.33409386
C	5.53904252	0.74158565	-0.09371594	H	-7.54375671	-3.78146853	0.73510333
C	5.70797018	3.37397549	2.55587323	C	-5.86812991	-2.43230272	0.71136012
H	5.09998272	3.72851912	3.38547719	H	-5.90126800	-2.29883627	-0.36675368
C	7.27427716	2.45464977	0.40901472	C	6.91437424	3.97542178	2.26638123
H	7.87701375	2.09756871	-0.42318415	H	7.26482664	4.81058252	2.86739166
C	4.87634409	-1.76853593	-1.45716619	C	7.70546333	3.51046464	1.18312740
C	4.86062612	-1.95431520	-2.85033447	H	8.65463951	3.99352883	0.96571490
H	4.12470443	-1.42490704	-3.45011712	C	-7.70546624	3.51046842	-1.18310844
C	5.79198649	-2.78681268	-3.47645247	H	-8.65464385	3.99352919	-0.96569469
H	5.76409028	-2.90835487	-4.55659072	C	-6.91437657	3.97543350	-2.26635840
C	6.76146541	-3.45050367	-2.72069848	H	-7.26482987	4.81059686	-2.86736462

Table S14: Cartesian coordinates of the *cisoid* optimized geometry of PAH **4a** at the B3LYP/6-31+G(d) level of theory**.**

C	2.26697336	-1.75163116	-0.11586811	C	-6.74817041	-2.84886074	-3.45521715
C	2.04875640	-3.12072376	-0.18654252	C	-6.79303819	-1.45950111	-3.30011175
C	0.76121117	-3.65601865	-0.39923509	C	-5.86313866	-0.81236530	-2.48551330
C	-0.32305152	-2.81883124	-0.49324432	C	4.33770745	-1.97377678	1.44335111
C	-0.17002746	-1.39923784	-0.47605592	C	5.53173241	-1.23412384	1.62136968
C	1.16200741	-0.84660979	-0.36665812	C	-6.51968956	1.52687469	2.57109051
C	-1.31688345	-0.55308920	-0.58258261	C	-5.11442566	3.39454513	3.24453501
C	-1.16200748	0.84660993	-0.36665858	C	-7.67030595	-0.46249316	1.77355500
C	0.17002742	1.39923791	-0.47605659	H	2.88550097	-3.79593479	-0.04152295
C	1.31688338	0.55308921	-0.58258282	H	0.62440320	-4.73418503	-0.42592453
C	-2.26697348	1.75163144	-0.11586907	H	-1.32025979	-3.24204790	-0.53754347
C	-2.04875644	3.12072402	-0.18654400	H	-2.88550098	3.79593513	-0.04152473
C	-0.76121116	3.65601875	-0.39923669	H	-0.62440314	4.73418512	-0.42592655
C	0.32305150	2.81883129	-0.49324552	H	1.32025976	3.24204791	-0.53754476

C	3.56622314	-1.27990588	0.38321123	H	2.32619118	1.91466138	-1.88424518
C	4.28908936	-0.16636164	-0.01802147	H	6.71700108	1.62587507	0.20818580
C	3.84573762	0.82204407	-0.98746789	H	3.21249169	-3.64852422	2.23166020
C	2.52911823	1.13219282	-1.15583989	H	4.93632031	-4.25093607	3.89006767
C	6.51968956	-1.52687360	2.57109108	H	8.51710449	1.14216738	1.82618768
C	5.55587496	-0.11950327	0.75101846	H	4.08232376	3.51582280	-1.44534562
C	6.63601748	0.74350611	0.83457320	H	5.72526370	4.66505327	-2.89283507
C	4.12268113	-3.05832901	2.27844113	H	7.47528514	3.35352188	-4.08626486
C	5.11442560	-3.39454369	3.24453640	H	7.55265355	0.87762545	-3.81642983
C	7.67030595	0.46249389	1.77355472	H	5.90283796	-0.26794459	-2.37288124
C	4.86090875	1.53732485	-1.81557466	H	-2.32619116	-1.91466202	-1.88424438
C	4.83013810	2.93348019	-1.97759542	H	-3.21249187	3.64852543	2.23165851
C	5.76352464	3.58341031	-2.78870976	H	-6.71700118	-1.62587486	0.20818638
C	6.74817089	2.84885818	-3.45521843	H	-4.08232341	-3.51582327	-1.44534344
C	6.79303829	1.45949867	-3.30011191	H	-5.72526296	-4.66505519	-2.89283221
C	5.86313850	0.81236370	-2.48551314	H	-7.47528450	-3.35352508	-4.08626330
C	-3.56622333	1.27990641	0.38321041	H	-7.55265345	-0.87762846	-3.81643025
C	-4.28908956	0.16636197	-0.01802184	H	-5.90283828	0.26794308	-2.37288228
C	-3.84573774	-0.82204417	-0.98746787	H	-4.93632036	4.25093774	3.89006596
C	-2.52911828	-1.13219304	-1.15583955	H	-8.51710438	-1.14216670	1.82618829
C	-5.55587507	0.11950377	0.75101828	C	6.28429078	-2.66666968	3.39455973
C	-4.33770760	1.97377764	1.44335013	H	7.01396582	-2.95441055	4.14811219
C	-4.12268128	3.05833019	2.27843974	C	7.62999127	-0.63395533	2.62033255
C	-6.63601754	-0.74350567	0.83457347	H	8.43750697	-0.80787548	3.32800300
C	-5.53173252	1.23412468	1.62136910	C	-7.62999122	0.63395637	2.62033243
C	-4.86090879	-1.53732574	-1.81557411	H	-8.43750686	0.80787671	3.32800289
C	-4.83013783	-2.93348122	-1.97759377	C	-6.28429078	2.66667108	3.39455873
C	-5.76352416	-3.58341216	-2.78870773	H	-7.01396572	2.95441217	4.14811117

6 References

1 M. M. Brahmi, J. Monot, M. Desage-El Murr, D. P. Curran, L. Fensterbank, E. Lacôte and M. Malacria, *J. Org. Chem.*, 2010, **75**, 6983.

2 G. R. Fulmer, A. J. M. Miller, N. H. Sherden, H. E. Gottlieb, A. Nudelman, B. M. Stoltz, J. E. Bercaw and K. I. Goldberg, *Organometallics*, 2010, **29**, 2176.

3 G. M. Sheldrick, *Acta Crystallogr. A Found. Adv.*, 2015, **71**, 3.

4 G. M. Sheldrick, *Acta Crystallogr. C Struct. Chem.*, 2015, **71**, 3.

5 Z. Chen, C. S. Wannere, C. Corminboeuf, R. Puchta and P. v. R. Schleyer, *Chem. Rev.*, 2005, **105**, 3842.

6 D. Geuenich, K. Hess, F. Köhler and R. Herges, *Chem. Rev.*, 2005, **105**, 3758.

7 M. J. Frisch, G. W. Trucks, H. B. Schlegel, G. E. Scuseria, M. A. Robb, J. R. Cheeseman, G. Scalmani, V. Barone, G. A. Petersson, H. Nakatsuji, X. Li, M. Caricato, A. V. Marenich, J. Bloino, B. G. Janesko, R. Gomperts, B. Mennucci, H. P. Hratchian, J. V. Ortiz, A. F. Izmaylov, J. L. Sonnenberg, Williams, F. Ding, F. Lipparini, F. Egidi, J. Goings, B. Peng, A. Petrone, T. Henderson, D. Ranasinghe, V. G. Zakrzewski, J. Gao, N. Rega, G. Zheng, W. Liang, M. Hada, M. Ehara, K. Toyota, R. Fukuda, J. Hasegawa, M. Ishida, T. Nakajima, Y. Honda, O. Kitao, H. Nakai, T. Vreven, K. Throssell, J. A. Montgomery Jr., J. E. Peralta, F. Ogliaro, M. J. Bearpark, J. J. Heyd, E. N. Brothers, K. N. Kudin, V. N. Staroverov, T. A. Keith, R. Kobayashi, J. Normand, K. Raghavachari, A. P. Rendell, J. C. Burant, S. S. Iyengar, J. Tomasi, M. Cossi, J. M. Millam, M. Klene, C. Adamo, R. Cammi, J. W. Ochterski, R. L. Martin, K. Morokuma, O. Farkas, J. B. Foresman and D. J. Fox, *Gaussian 16 Rev. C.01*, Wallingford, CT, 2016.

8 A. Rahalkar, A. Stanger "Aroma", *http://chemistry.technion.ac.il/members/amnon-stanger/*.
9 A. Stanger, *J. Org. Chem.*, 2006, **71**, 883.
10 A. Stanger, *J. Org. Chem.*, 2010, **75**, 2281.
11 R. Gershoni-Poranne and A. Stanger, *Chem. Eur. J.*, 2014, **20**, 5673.
12 M. J. Frisch, G. W. Trucks, H. B. Schlegel, G. E. Scuseria, M. A. Robb, J. R. Cheeseman, G. Scalmani, V. Barone, B. Mennucci, G. A. Petersson, H. Nakatsuji, M. Caricato, X. Li, H. P. Hratchian, A. F. Izmaylov, J. Bloino, G. Zheng, J. L. Sonnenberg, M. Hada, M. Ehara, K. Toyota, R. Fukuda, J. Hasegawa, M. Ishida, T. Nakajima, Y. Honda, O. Kitao, H. Nakai, T. Vreven, J. A. Montgomery, J. E. Peralta, F. Ogliaro, M. Bearpark, J. J. Heyd, E. Brothers, K. N. Kudin, V. N. Staroverov, R. Kobayashi, J. Normand, K. Raghavachari, A. Rendell, J. C. Burant, S. S. Iyengar, J. Tomasi, M. Cossi, N. Rega, J. M. Millam, M. Klene, J. E. Knox, J. B. Cross, V. Bakken, C. Adamo, J. Jaramillo, R. Gomperts, R. E. Stratmann, O. Yazyev, A. J. Austin, R. Cammi, C. Pomelli, J. W. Ochterski, R. L. Martin, K. Morokuma, V. G. Zakrzewski, G. A. Voth, P. Salvador, J. J. Dannenberg, S. Dapprich, A. D. Daniels, Ö. Farkas, J. B. Foresman, J. V. Ortiz, J. Cioslowski and D. J. Fox, *Gaussian 09 Revision A.2*, 2009.
13 T. Lu and F. Chen, *J. Comput. Chem.*, 2012, **33**, 580.
14 A. D. Becke, *J. Chem. Phys.*, 1988, **88**, 2547.
15 M. Yamashita, K. Kawano, A. Matsumoto, N. Aratani, H. Hayashi, M. Suzuki, L. Zhang, A. L. Briseno and H. Yamada, *Chem. Eur. J.*, 2017, **23**, 15002.
